# Genome-Wide Identification and Analysis of Anthocyanidin Reductase Gene Family in Lychee (*Litchi chinensis* Sonn.)

**DOI:** 10.3390/genes15060757

**Published:** 2024-06-08

**Authors:** Bin Liang, Xiuxu Ye, Huanling Li, Fang Li, Shujun Wang, Chengdong Jiang, Jiabao Wang, Peng Wang

**Affiliations:** 1 College of Tropical Agriculture and Forestry, Hainan University, Danzhou 571737, China; 2 Institute of Environment and Plant Protection, Chinese Academy of Tropical Agricultural Sciences, Haikou 571101, China; 3 Institute of Tropical Crop Genetic Resources, Chinese Academy of Tropical Agricultural Sciences, Haikou 571101, China

**Keywords:** lychee, pericarp coloring, anthocyanidin reductase, gene family, gene expression, heterologous gene expression

## Abstract

Anthocyanidin reductase (*ANR*) is a key enzyme regulating anthocyanin synthesis and accumulation in plants. Here, lychee *ANR* genes were globally identified, their sequence and phylogenetic characteristics were analyzed, and their spatiotemporal expression patterns were characterized. A total of 51 *ANR* family members were identified in the lychee genome. The length of the encoded amino acid residues ranged from 87 aa to 289 aa, the molecular weight ranged from 9.49 KD to 32.40 KD, and the isoelectric point (pI) ranged from 4.83 to 9.33. Most of the members were acidic proteins. Most members of the *LcANR* family were located in the cytoplasm. The 51 *LcANR* family members were unevenly distributed in 11 chromosomes, and their exons and motif conserved structures were significantly different from each other. Promoters in over 90% of *LcANR* members contained anaerobically induced response elements, and 88% contained photoresponsive elements. Most *LcANR* family members had low expression in nine lychee tissues and organs (root, young leaf, bud, female flower, male flower, pericarp, pulp, seed, and calli), and some members showed tissue-specific expression patterns. The expression of one gene, *LITCHI029356.m1*, decreased with the increase of anthocyanin accumulation in ‘Feizixiao’ and ‘Ziniangxi’ pericarp, which was negatively correlated with pericarp coloring. The identified *LcANR* gene was heterologously expressed in tobacco K326, and the function of the *LcANR* gene was verified. This study provides a basis for the further study of *LcANR* function, particularly the role in lychee pericarp coloration.

## 1. Introduction

Lychee (*Litchi chinensis* Sonn.), also named litchi or leechee, is an important fruit crop in tropical and subtropical regions around the world. This delicate fruit is cherished for its succulent edible portion and it plays a pivotal role in the local economy [1] of countries such as China [2], India, and Thailand, where it is widely cultivated and forms an integral part of both domestic consumption and international trade. For example, there were 527,000 hectares lychee in 2023In China [3]. Lychee cultivation contributes enormously to the livelihood of many small-scale farmers and agribusinesses, making it a vital crop with far-reaching economic and cultural significance.

The pericarp color of a lychee is one of the key traits from the consumer perspective [4]. Customers often make a purchasing decision based on the visual appeal of the pericarp, and thus the pericarp color often influences shopping behavior, causing participants in the fruit supply chain to associate rich pericarp colors with the optimal commercial value of a particular fruit [5]. For lychees, the trait of red pericarp serves as a key visual cue for consumers [6]. This identifiable and appealing hue is often associated with a specific variety and can be a mark of higher quality fruit; thus, it is often used by retailers as a standard to attract customers, making it a highly coveted trait in lychee cultivation. Furthermore, the appeal of the red pericarp color extends into the realm of lychee agricultural practices and breeding programs. In the field of lychee breeding, predecessors have conducted research on improving the coloring rate of lychees and making the fruit color bright red by spraying exogenous hormones [7,8,9], changing lighting [10,11] or temperature [12], and achieving changes in the color of lychee skin after ripening.

The red pericarp color of a lychee is primarily a result of the anthocyanin accumulation in the pericarps, which is what gives the fruit its distinctive appearance [13]. Anthocyanins are a group of pigments responsible for the red, purple, and blue colors in many fruits and vegetables. However, the specific mechanisms and factors that regulate the biosynthesis and accumulation of anthocyanins in the lychee pericarp remain largely unknown. In fact, the mechanisms underlying anthocyanin synthesis have been the subject of extensive study in model plants and other fruit crops. The biosynthetic pathway of anthocyanins involves a series of enzymatic reactions that convert simple molecules into complex pigments [14,15,16]. Researchers have identified key regulatory genes and enzymes involved in this process, shedding light on how plants produce and accumulate anthocyanins in response to environmental cues and developmental signals. It was found that chalcone synthase (CHS) [17] is the first key enzyme in anthocyanin synthesis pathway [15,18], followed by naringin chalcone isomerase (CHI), flavanone 3-hydroxylase (F3H), dihydroflavonol-4-reductase (DFR), and anthocyanidin synthase (ANS) under continuous action to form anthocyanins [19,20], while anthocyanidin reductase (*ANR*) [21] is the key enzyme for the synthesis of proanthocyanidin (PC). PC can be generated under the catalysis of *ANR* after the formation of anthocyanin. UFGT can catalyze anthocyanins to stable anthocyanins with glycosides. Stable anthocyanins with glycosides cannot be catalyzed by *ANR* to generate PC [22,23]. In fact, *ANR* plays a negative regulatory role in anthocyanin biosynthesis. Studies have shown that the absence of *ANR* promotes anthocyanin synthesis and increases anthocyanin content in Arabidopsis [24,25]. The *ANR* gene has been identified in tea tree [26,27,28], blueberry [29], apple [30], grape [31,32,33], peach [34], mango [35], and safflower [36], among other plants. In the study on the postharvest physiology of lychee, laccase was found to play a catalytic role in pericarp browning [37]. The substrate for pericarp browning is PC [38,39,40]. Therefore, the activity of *ANR* affects the degree of pericarp browning after fruit picking [41,42]. In the previous study of pericarp browning, five *ANR* family members were identified in lychee [43,44]. However, the *ANR* family members in lychee have not been studied in detail.

The FZX and ZNX used for genome assembly are known diploid lychees. In this study, the lychee *ANR* family members were comprehensively identified from the lychee reference genome. A total of three segmental-duplicated gene pairs with six *LcANR* genes were also identified on lychee chromosomes (*LITCHI014912.m1*&*LITCHI003640.m1*, *LITCHI022853.m1*&*LITCHI025537.m1*, *LITCHI013339.m1*&*LITCHI029352.m1*). The sequence and protein physicochemical characteristics were analyzed. The expression pattern in different lychee tissues, as well as in the pericarp of two lychee varieties at different coloring stage, was investigated. These results provide a basis for research on lychee pericarp coloring and the function of *ANR* genes.

## 2. Materials and Methods

### 2.1. Experimental Materials

**Figure 1:**
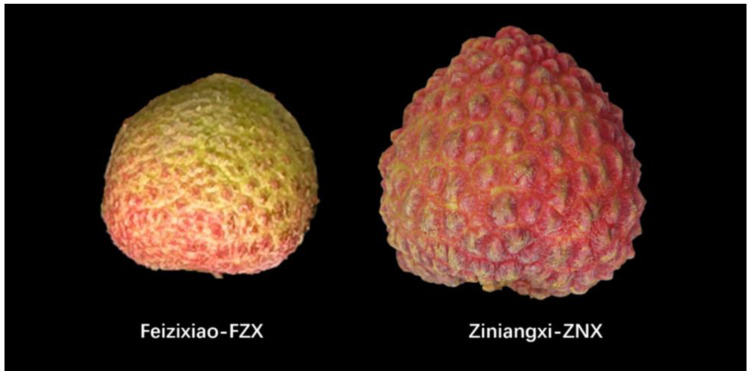
‘Feizixiao’ (F9) and ‘Ziniangxi’ (Z12) fruit at harvest maturity stage.

From March to June in 2020 and 2023, the fruits of the ‘Feizixiao’ (FZX) and ‘Ziniangxi’ (ZNX) lychee tree were collected at Yongfa Lychee Research Demonstration Base of Tropical Fruit Research Institute of Hainan Academy of Agricultural Sciences. We chose these two varieties because of their color at harvest maturity: FZX was poorly colored while ZNX was almost completely red (Figure 1). The two varieties had nearly the same flowering time. The fruit samples were collected once a week, beginning on the 10th day after full blooming (DAB). Twenty similar fruits were selected at 10th DAB as reference fruit. Samples were collected every 7 days. Only fruits that were similar in size to the reference fruit were collected. The fruit samples were shipped back to the laboratory within 2 h of collection. They were carefully separated into pericarp, seeds, and pulp, and each part was frozen in liquid nitrogen and then stored at −80 ℃ for further use. Twenty fruits were set as a repeat, and three repeats were set for each sampling. In this research, we named the sampling date of 10th DAB of ZNX and FZX as Z1 and F1, respectively. The subsequent samples were named as Z2, F2, and so on. FZX fruit ripened at F9 stage, and ZNX fruit ripened at Z12 stage.

In March 2020, the buds and young leaves of spring branches, female flowers, male flowers, and the white roots of air-layering seedlings of FZX lychee were picked at the same place. The embryonic calli, induced and preserved as described by Wang [45], were also sampled. These materials were frozen in liquid nitrogen immediately after sampling and then stored at −80 °C for further use.

### 2.2. Identification and Physicochemical Properties of Anthocyanidin Reductase Gene Family in Lychee Genome

First, we found the published *ANR* gene (AT1G61720) [24,25] in Arabidopsis thaliana, and we found the conserved domain of *ANR* (PF01370) in the Arabidopsis genome database (https://www.arabidopsis.org/, accessed on 11 July 2023). The whole genome protein sequence of lychee was downloaded from the whole genome database of lychee. The lychee *ANR* gene ID containing (PF01370) was found from the data downloaded from the lychee genome database. In addition, the gene sequences of lychee *ANR* family members and lychee gff3 files were downloaded from the lychee genome database.

The lychee protein sequence was derived from the lychee genome database [46] (http://www.sapindaceae.com/, accessed on 12 July 2023). The hidden Markov model (PF01370) was obtained by Arabidopsis *ANR* [24] in Pfam (http://pfam.xfam.org/, accessed on 12 July 2023). The HMMER3.0 program was used to search the lychee protein sequence in the database to obtain candidate genes for preliminary screening. SMART (https://smart.embl-heidelberg.de/, accessed on 13 July 2023). ExPASy (https://www.expasy.org/, accessed on 13 July 2023) was used to analyze the physical and chemical properties of *LcANR* family members. Molecular Bioinformatics Center *(*http://cello.life.nctu.edu.tw, accessed on 13 July 2023) was used to predict subcellular localization and to predict the candidate gene family.

### 2.3. Amino Acid Sequence Alignment, Gene Structure Analysis, and Chromosome Localization of Lychee ANR Family Members

The *LcANR* family protein sequence from the lychee genome database [46] (https://data.mendeley.com/datasets/kggzfwpdr9/1, accessed on 7 August 2023) was obtained from the lychee genome for amino acid lychee genome database sequence alignment. The gene structure analysis of the *ANR* family in lychee was obtained by downloading the gff3 file from the lychee gene database and visualizing it by using the TBtools software analysis tool. TBtools and (http://meme-suite.org/, accessed on 8 August 2023) were used to analyze the domain of the *LcANR* family (https://tbtools.updatestar.com/, accessed on 8 August 2023). Map Chart Creator V2.0 (https://mapchart.net/, accessed on 10 August 2023) was used for chromosome localization analysis. Properties of *ANR* family members in litchi are shown in Table S1.

### 2.4. Construction of Lychee Genome LcANR Family Phylogenetic Tree

The whole protein sequence of Arabidopsis thaliana was downloaded from the Arabidopsis genome database (https://www.arabidopsis.org/, accessed on 20 August 2023), and the Apple genome database (http://bioinfo.bti.cornell.edu/ftp/Apple_genome/genome/haploid/Gala/, accessed on 21 August 2023); tea plant and grape information was obtained from NCBI (http://www.ncbi.nlm.nih.gov/, accessed on 22 August 2023). The *ANR* gene ID containing (PF01370) was found from the data of the whole genome protein sequence of these species. The protein sequences used to construct the evolutionary tree include 38 Arabidopsis *ANR* family members, 83 tea *ANR* family members, 68 apple *ANR* family members, 57 grape *ANR* family members, and 51 lychee *ANR* family members. The genes with the same domain as those identified in lychee were further analyzed by near-source species and model plants. In this phylogenetic tree, the branch of Arabidopsis *ANR* gene AT1G61720, which has been reported many times [24,25], was extracted to further determine the function of the *LcANR* gene in lychee. MUSCLEv3.8.31 was used to construct the *ANR* family phylogenetic tree of lychee, tea, apple, grape, and Arabidopsis.

### 2.5. Analysis of Cis-Acting Elements in Lychee LcANR

To understand the structure of *LcANR* genes, the online program MEME (https://meme-suite.org/meme/tools/meme, accessed on 18 September 2023) was commonly applied to analyze the conserved motifs. We downloaded domains from the NCBI Conserved Domain Database. The online website PlantCARE (http://bioinformatics.psb.ugent.be/webtools/plantcare/html/, accessed on 21 September 2023) was applied to predict the cis-acting elements within 2000 bp upstream of all *LcANR* genes [47]. We obtained intron/exon structure information from the lychee gff3 file. genes [46]. The visualization was performed with TBtools software.

### 2.6. Expression Analysis of Lychee ANR Family Members in the Lychee Transcriptomes

In our ongoing research, we used transcriptome data from various tissues, including pericarp at Z2, Z5, Z8, Z12, F2, F5, and F9 stage, and tissues of FZX such as seed, pulp, pericarp from matured fruit, leaves, buds, male flower, female flower, white roots of air-layering seedlings, and embryonic calli. The expression patterns of the *ANR* gene family were obtained by analyzing the transcriptome data and confirmed by qRT-PCR with random selected genes. Each sample had 3 biological replicates and 3 technical replicates.

As for qRT-PCR, total RNA was extracted from the above tissues using a rapid universal plant RNA extraction kit (Huachiu Ocean Co., Ltd., Beijing, China) and were reverse-transcribed into cDNA using a reverse transcription kit (TaKaRa Bio, Beijing, China). Nine genes with different transcriptome expression trends were selected for qRT-PCR experiments. According to the coding region sequence of the gene, the primers of the gene were designed by Primer 5.0. The primer information was sent to Shenggong Bioengineering Co., Ltd. (Shanghai, China) for synthesis. The primer information is shown in Table S2.

The qRT-PCR experiments were performed using a fluorescent quantitative PCR instrument (Thermo Fisher Scientific, Waltham MA, USA). The qRT-PCR reaction solution was prepared according to the instructions for use by TB Green Premix Ex TaqII (TaKaRa Bio, Beijing, China). The procedure was as follows: pre-denaturation at 95 °C for 20 s, denaturation at 95 °C for 5 s, and annealing at 60 °C for 20 s. The cycle was performed 40 times, and the melting curve stages were 95 °C for 15 s, 60 °C for 60 s, and 95 °C for 15 s for one cycle. The reaction system was as follows: TB Green Premix Ex TaqII (2×) 12.5 μL, PCR Primers (10 μmol/L) 1 μL, RT reaction solution (cDNA solution) 2 μL, and ddH_2_O 8.5 μL. The total volume was 20 μL. The Ct value of ‘Feizixiao’ lychee bud was set as 1, and the relative expression was calculated using the 2^−△△Ct^ method. The expression levels of the *ANR* gene in 9 tissues and the organs of a lychee were analyzed. The Ct value of the pericarp at Z2 stage was set as 1, and the relative expression level was calculated using the 2^−△△Ct^ method. The expression of the selected 9 genes in different fruit pericarps of ZNX and FZX was analyzed.

### 2.7. Determination of Total Anthocyanins in the Pericarps of ‘Feizixiao’ and ‘Ziniangxi’ Lychee

The frozen pericarp of the FZX and ZNX lychee was used as the material. A total of 0.5 g liquid nitrogen powder of the pericarp was taken into the ice pre-cooled mortar, and an appropriate amount of methanol solution containing 1% of HCl was added to continue grinding into the homogenization. The homogenization liquid was transferred into the glass test tube and kept under dark light for 2 h, then filtered, and the volume was fixed at 25 mL. The absorbance at 600 nm and 530 nm was determined by the UV spectrophotometer (UV2450, Shimadzu, Japan). The anthocyanin content was calculated as follows: anthocyanin content (0.01ΔA/gFW) = 100 × (A530 − A600)/fresh weight of sample. The determination of proanthocyanidins in the pericarps of the FZX and ZNX lychee was performed by Entrust Maiwei Company (Wuhan, China). Each sample had 3 biological replicates and 3 technical replicates.

### 2.8. Cloning of LcANR(LITCHI029356.m1) Gene and Construction of LcANR-OE Vector

Total RNA was extracted from the pericarp of the FZX lychee using a rapid universal plant RNA extraction kit (Huachiu Ocean Co., Ltd., Beijing, China) and reverse transcribed into cDNA using a reverse transcription kit (TaKaRa Bio, Beijing, China). Nine genes with different transcriptome expression trends were selected for qRT-PCR experiments. According to the coding region sequence of the gene, the primers of the gene were designed by Primer 5.0. The primer information was sent to Shenggong Bioengineering Co., Ltd. (Shanghai, China) for synthesis (primer sequence: *LcANR-CDS-F*: ATGGCCAGCGAGTTCACCG *LcANR-CDS-R*: TCACTTAAGCAGCCCCCTAGT). The sequencing results were compared with the gene (http://www.sapindaceae.com/, accessed on 7 September 2023) sequence, and the sequence was consistent with the description of the target gene. The vector was constructed by the TA cloning (CV17-Zero Background pTOPO-Blunt Simple Cloning Kit, Aidlab, Beijing, China) experiment. After the vector was transferred into *E. coli*, the bacterial solution was sent for sequencing to verify the cloning of the target gene.

The electrophoresis fragment of *LcANR-CDS* with a length of 1008 bp was cut off, and the target fragment was recovered by agarose gel DNA recovery kit (Huachiu Ocean, Beijing, China). In the transgenic experiment, the name of the transgenic vector we used was pCAMBIA1300, and the promoter in the vector was 35 S. The PCR detection band size was about 1100 bp. The positive plaque was detected, and the shaking bacteria were picked up. Sanger sequencing was performed on the bacterial solution, and the sequencing was entrusted to Bioengineering (Shanghai, China) Co., Ltd. The sequencing primer eGFP-cx was used to test the sequence, and the sequencing results were consistent with the sequence of the target fragment, that is, the overexpression vector was successfully constructed. The primer information is shown in Table S3. Vector map and vector sequence are shown in Table S4.

### 2.9. Construction of ANR-RNAi Vector

Knockdown *NtANR*(*XM_016589997.1*) in tobacco, which is the homologous gene most similar to the lychee *LcANR*(*LITCHI029356.m1*) sequence, further indicates that the similarity of gene structure will lead to the same function, which proves the function of *LcANR*. The homologous gene sequence of *LcANR*(*LITCHI029356.m1*) in tobacco was found using a database (http://www.sapindaceae.com/, accessed on 10 September 2023), and the homologous gene with high homology of *LcANR* in lychee was found for sequence alignment. The homologous genes of tobacco and the genes with a high similarity to the *LcANR* sequence were compared in NCBI to exclude interference with other genes. The results were compared and analyzed, and the *NtANR*(*XM_016589997.1*) gene in tobacco was the most similar to the *LcANR*(*LITCHI029356.m1*) sequence to design specific interference fragments. In the transgenic experiment, the name of the transgenic vector we used was pCAMBIA1300, and the promoter in the vector was 35 S. The target fragment was amplified by PCR to obtain the fragment ligation vector. The 288 bp electrophoresis fragment (ATATTTTTGTTGCTGAGAAAGAATCAGCTTCCGGACGATACATTTGTTCTGCCATCAACACCAGTGTTCCGGAGCTAGCAAATTTCTTGAAGAAAAGATATCCAACTTCGGATGTTCCTACAGATTTCGGGGATTTCCCCTCCAAGGCCAAGTTGATCATCTCGTCAGAAAAGCTCATCAAAGAGGGATTCAATTTTAAGTATGGGATTGAAGAGATTTATGATCAATGTCTTGCTTGTTTTAAAGATAAGGGGTTACTAAAGAACTGAATAGTTCAGTTAATATTGT) was cut off and the target fragment was recovered using the agarose gel DNA recovery kit (Huachiu Ocean, Beijing, China). The primers of Linker (+)/NOS-R were used for colony PCR detection, and a band of about 490 bp was obtained. The positive plaque was detected, and the single colony shaking bacteria were picked. The plasmid extracted from the above-mentioned positive plaque was entrusted to Shenggong Bioengineering (Shanghai, China) Co., Ltd., and the sequencing primer Linker (+)/Linker (−) was used for bidirectional tests. The sequencing result sequence was consistent with the target fragment sequence alignment, that is, the overexpression vector was successfully constructed. The primer information is shown in Table S5. The vector map and vector sequence are shown in Table S6.

### 2.10. Genetic Transformation of LcANR Gene in Tobacco

The method of transferring *LcANR*(*LITCHI029356.m1*)into tobacco mainly refers to the method of transferring an apple gene into tobacco by HAN [48]. Tobacco K326 seeds were sterilized and sowed on MS or 1/2 MS medium, and the leaves at the 3–4 leaf stage were grown to obtain sterile leaves as explants. The explants prepared by resuscitating the bacteria for 1–2 h with the Agrobacterium suspension containing the constructed vector were placed in the Agrobacterium suspension. After 10 min of infection, the Agrobacterium liquid on the surface of the explants was dried with sterile filter paper. The dried tobacco explants were placed in co-culture medium at 25 °C for 2 days in the dark. The differentiation and screening of tobacco genetic transformation seedlings were carried out at the same time, and the differentiation time was 30–40 d. A DNA extraction kit (Huachiu Ocean Co., Ltd., Beijing, China) was used to extract DNA from tobacco leaves, and a conventional PCR amplification was used to detect whether tobacco seedlings contained the hygromycin gene, which verified that *LcANR*(*LITCHI029356.m1*) was successfully transformed into tobacco. The primers for the hygromycin gene detection were as follows: (H (+): GCCAACCCGGCCTCCAGAAGA; H (−): CCGCAAGGAAATCGGTCAATACA). The band size of the positive material in the test results was 307 bp.

## 3. Results

### 3.1. Phylogeny of LcANR Sequences

**Figure 2:**
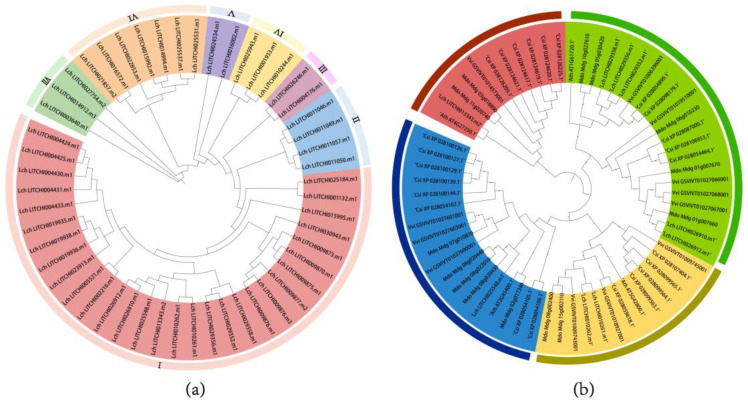
Phylogeny of *LcANR* Sequences: (**a**) phylogenetic tree of *ANR* family in lychee; (**b**) partial branches of *ANR* family phylogenetic tree for Arabidopsis, tea, apple, grape and lychee.

Phylogenetic relationships were inferred for the lychee *ANR* peptide sequences, which showed that *LcANR* can be divided into seven subgroups, namely subgroups Ⅰ, Ⅱ, Ⅲ, Ⅳ, Ⅴ, Ⅵ, and Ⅶ. Subgroup Ⅰ was the largest, containing 30 *LcANR* family members, while subgroups Ⅱ and Ⅵ had 4 and 7 family members, respectively. Subgroup Ⅳ and subgroup Ⅶ had 3 *LcANR* family members. Subgroups Ⅲ and Ⅴ had 2 *LcANR* family members, respectively (Figure 2).

A phylogenetic tree of the *ANR* family of lychee, tea plant, apple, grape, and Arabidopsis was reconstructed using 38 *ANR* family protein sequences of Arabidopsis, together with 83 tea tree, 68 apple, 57 grape, and 51 lychee ones. In this tree, the branch of Arabidopsis *ANR* gene *AT1G61720*, which had been reported [24,25], was extracted to further determine the function of the *LcANR* gene in Lychee. *LITCHI029356.m1* was found to be in the same branch as that of the Arabidopsis *ANR* gene, and the relative expression results obtained by high-throughput sequencing data and q-PCR were consistent with the anthocyanin content measured in lychee pericarp (F). These results indicated that *LITCHI029356.m1* might function as anthocyanidin reductase in lychee.

### 3.2. Identification and Sequence Characterization of Lychee ANR Family Members

In this study, to identify *LcANR* family genes in lychee, the gene sequences of the Arabidopsis *ANR* family were used to perform the genome-wide PFAM and HMME search using the lychee database. A total of 51 *ANR* family members were identified in the lychee genome. The amino acid sequence length, molecular weight, isoelectric point, and subcellular localization of the identified genes were predicted and analyzed. The results are shown in Table S1. The length of the *ANR* protein is between 87 and 289 amino acids, and the molecular weight is between 9.49 and 32.40 KD. The isoelectric points (pI) of the 40 family members were all below 7, indicating that most of the Lc *ANR* proteins were acidic proteins. Prediction analysis showed that 40 members were located in the cytoplasm, 8 in the periplasm, and the remaining members were located in the outer membrane of the cell membrane, indicating that the lychee *ANR* family mainly functions in the cytoplasm.

### 3.3. ANR Motifs and Gene Structure

By analyzing the gene structure and motif of *LcANR* family members, it was found that the number of exons among *LcANR* family members was quite different. The motif analysis showed that the motifs of each member, composed of 1–9 motifs, were also quite different. Among them, motifs 1, 2, 3, 4, 5, and 7 appeared most in the lychee *ANR* family. Motif 1 appeared the most in the N-terminus, and motif 5 appeared the most in the C-terminus. Most of the genes have a similar motif structure. According to the similarity of motif distribution, lychee *ANR* members can be divided into 6 sub-branches (Figure 2a,b).

**Figure 3:**
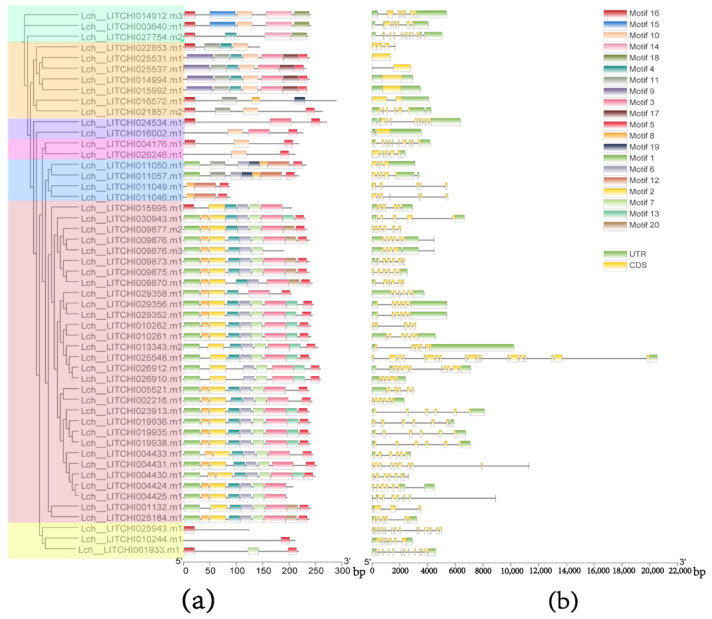

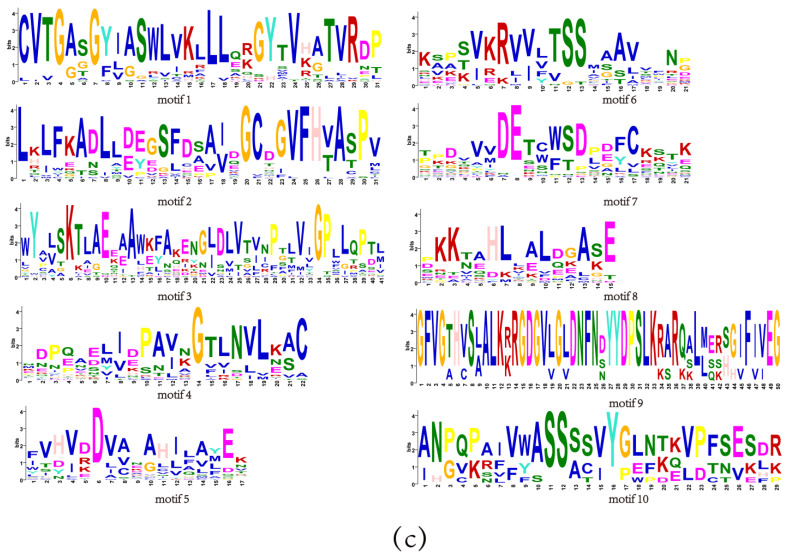
Identification and Sequence Characterization of Lychee *ANR* Family Members: (**a**) motifs of lychee *ANR* genes, different colors in the shadow represent different subfamilies; (**b**) exon-intron structure of lychee *ANR* genes; (**c**) top 10 conserved motifs in lychee *ANR* peptides.

The results of the motif atlas showed that motif 5 contained the most conserved residues, which were composed of 17 amino acid sites. The 6th position in motif 5 is a conserved amino acid site, corresponding to aspartic acid residue (Figure 3c).

### 3.4. Chromosome Distribution, Tandem Gene Duplication, and Segmental Gene Duplication of ANR Genes in Lychee

**Figure 4:**
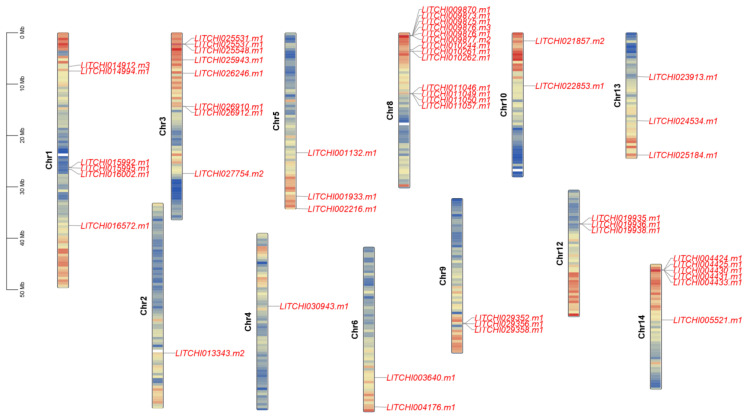
Chromosomal localization of *ANR* family members in lychee.

The *LcANR* family members are distributed on 11 chromosomes and are unevenly distributed, namely Chr1, Chr3, Chr4, Chr5, Chr6, Chr8, Chr9, Chr10, Chr12, Chr13, and Chr14. Most members are located on Chr8. There are 12 family members on Chr8. There are eight family members on Chr13. In contrast, only one member is located on Chr4 (Figure 4).

**Figure 5:**
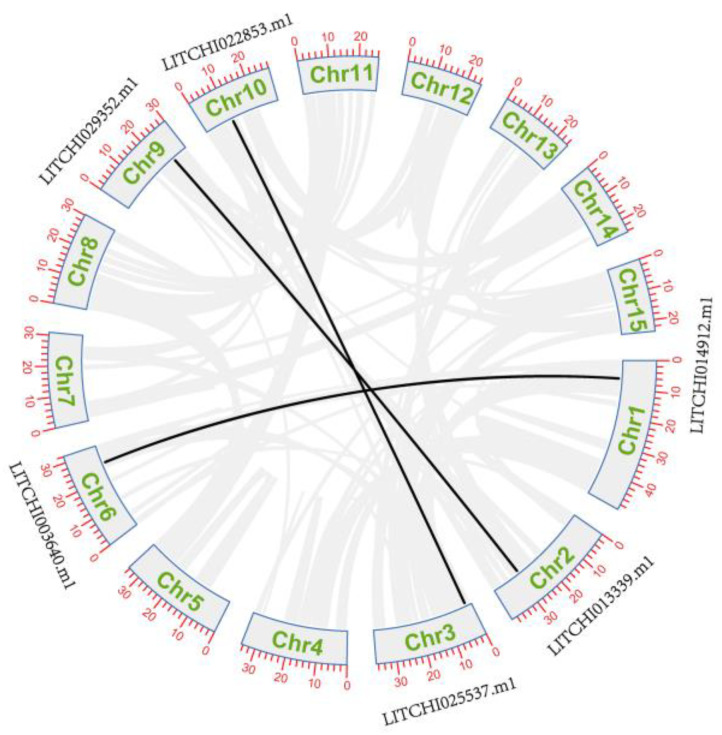
Segmental-duplication pairs of *LcANR* genes as indicated by black lines.

To further understand the expansion of the *LcANR* gene family, we also analyzed segmental-duplicated gene pairs. Three segmental-duplicated gene pairs with six *LcANR* genes were also identified on lychee chromosomes (*LITCHI014912.m1*&*LITCHI003640.m1*, *LITCHI022853.m1*&*LITCHI025537.m1*, *LITCHI013339.m1*&*LITCHI029352.m1*) (Figure 5).

### 3.5. Cis-Acting Elements of ANR Family Members in Lychee

**Figure 6:**
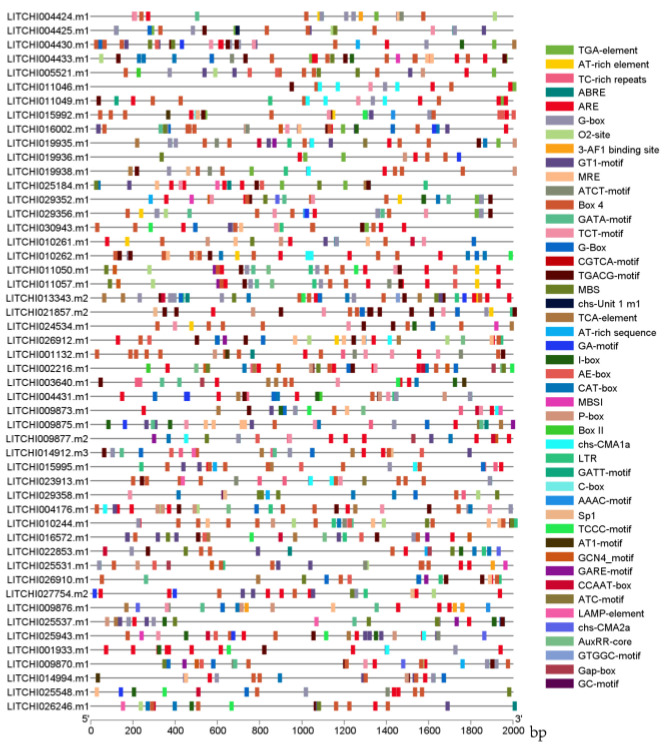
Distribution of cis-acting elements of lychee *ANR* genes.

To understand the promoter sequence function of *LcANR* family members, we analyzed DNA sequences 2000 bp at the upstream of ORF (open reading frame) sequence of 51 *LcANR* family members (Figure 6). The cis-acting elements of the *LcANR*s promoters were predicted using the PlantCARE online tool [47]. The results showed that only the gene *LITCHI009876.m3* had TATA-box and CAAT-box, and the other 50 family genes had TATA-box and CAAT-box.

Over 90% of the *ANR* members contain anaerobic-induced response elements, indicating that lychee *ANR* family members may play an important role in the redox process [49]. Over 88% of the members contain photoresponsivity elements, indicating that the gene expression of *LcANR* family may be regulated by light. Roughly 80% of the family members participate in abscisic acid reaction; 24 members participate in drought induction and 47% participate in salicylic acid reaction, suggesting that the *ANR* family members play an important role in resistance to adverse environment in lychee (Table 1).

### 3.6. Expression Analysis of ANR Family Members in Lychee

**Figure 7:**
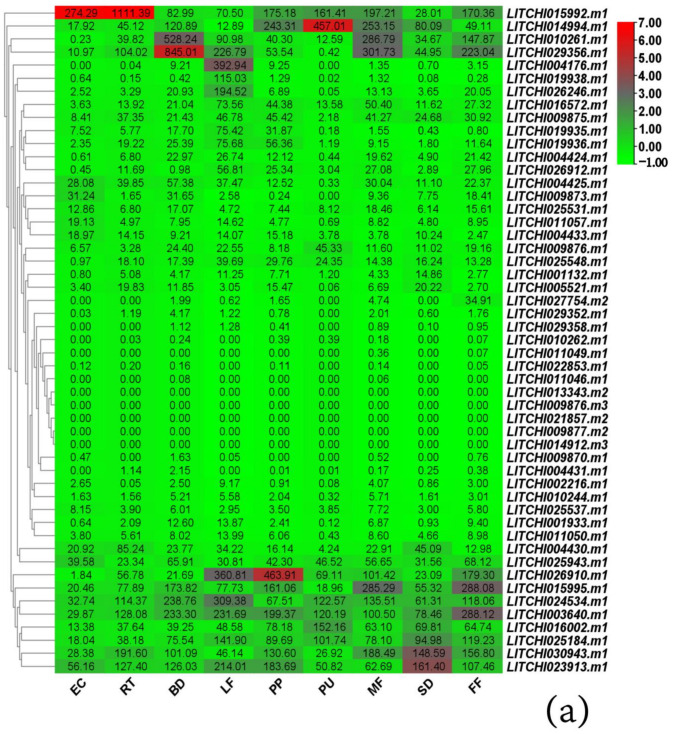

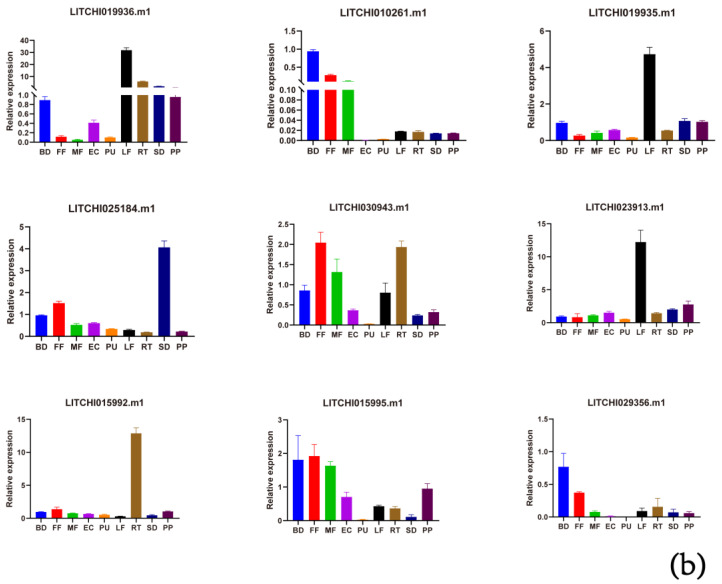
Expression Analysis of *ANR* Family Members in Lychee: (**a**) expression patterns of *ANR* family genes in nine tissues and organs of ‘Feizixiao’ lychee; (**b**) relative expression levels of *ANR* family genes in nine tissues and organs of ‘Feizixiao’ lychee. Each sample had three biological replicates and three technical replicates. The numbers in the Figure represent the FPKM values of gene expression levels. MF: Male flowers; PU: Fruit pulp; EC: Embryonic calli tissue; RT: Root; LF: Young leaves; BD: Bud; SD: Seed; FF: Female flowers; PP: Fruit harvesting period Peel. Data were presented as a mean (±SE) from three independent biological replicates.

The expression of 51 *LcANR* family members was analyzed in nine tissues and organs, i.e., white root of air-layering seedlings, young leaf, bud, female flower, male flower, pericarp, pulp, seed, and embryonic calli. The results showed that the expression patterns of 51 genes were divided into four types. The first was with no expression, that is, the FPKM value was 0. The second showed low expression with the FPKM value ranged between 0 and 50. The third was moderate expression pattern with the FPKM value ranged from 50 to 200, and the fourth was high expression with the FPKM value over 200. A total of 5 genes had no gene expression, 25 had low gene expression, 11 had moderate gene expression, and 11 had high gene expression in different tissues. Among them, the *LITCHI029356.m1* gene was highly expressed in buds, stamens, and pistils (Figure 7a).

RT-qPCR was used to analyze the relative expression levels of nine members of the *LcANR* family in different tissues and organs. The results were similar to those of transcriptome analysis. For example, in the results of RT-qPCR analysis, the expression level of *LITCHI015992.m1* in the root was also highly expressed. The expression level of *LITCHI019935.m1* in the transcriptome data was the highest in leaves, followed by peel and bud, and the same results were obtained in RT-qPCR analysis (Figure 7b).

### 3.7. Expression Analysis of LcANR Family Members in Lychee Pericarps

**Figure 8:**
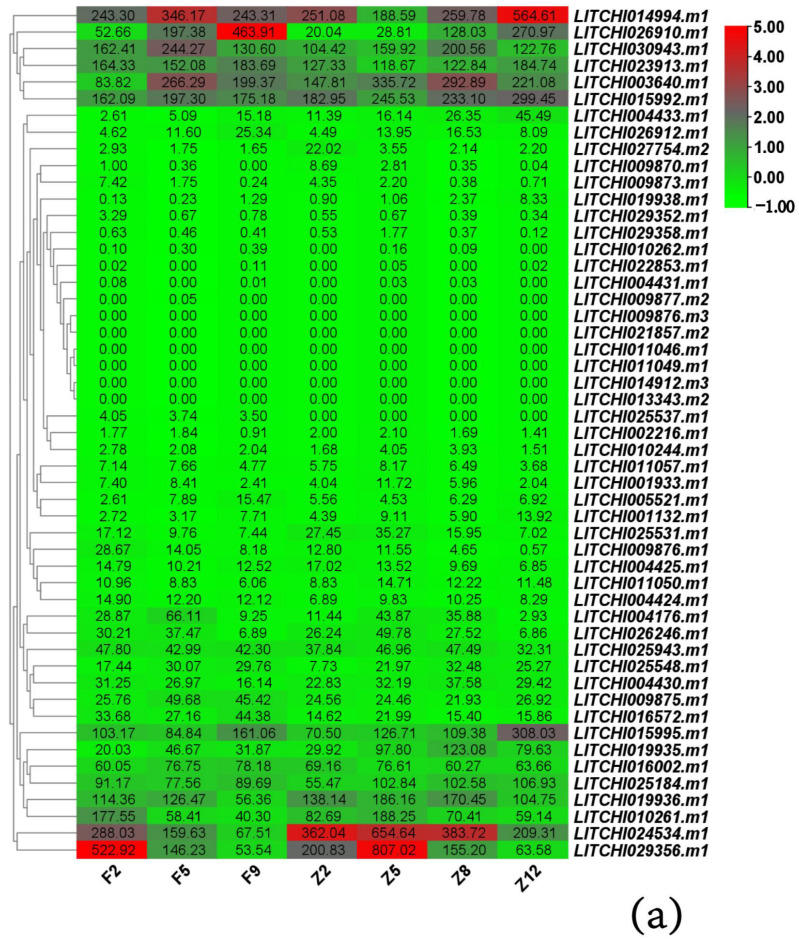

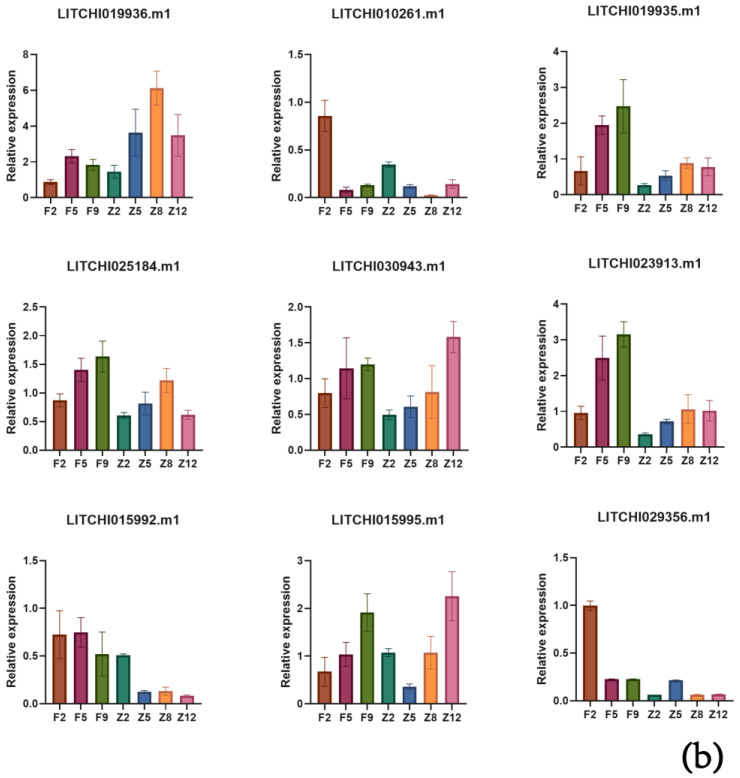
Expression Analysis of *LcANR* Family Members in lychee pericarps: (**a**) expression patterns of *LcANR* family genes in lychee pericarp of FZX and ZNX revealed by RNA-Seq; (**b**) the relative expression levels of randomly selected *LcANR* genes in the pericarps of FZX and ZNX lychees revealed by qRT-PCR. Each sample had three biological replicates and three technical replicates. The numbers in the figure represent the FPKM values of gene expression levels. F2, F5, F9, Z2, Z5, Z8, and Z12 represent the sampling stage of FZX and ZNX. Data were presented as a mean (±SE) from three independent biological replicates.

The expression of *LcANR* family members in lychee pericarps of two lychee varieties, FZX and ZNX, was investigated. According to the classification described in Section 3.7, 6 genes were not expressed, 31 had low expression, 6 had medium expression, and 8 had high expression in the fruit pericarp at different stages. The expression levels of the *LITCHI029356.m1* gene were high in pericarps of both FZX and ZNX, and the expression level gradually decreases with the coloring of the pericarp in both varieties (Figure 8a).

The relative expression level of nine members of the lychee *ANR* family in different tissues and organs was confirmed with RT-qPCR, which showed that the results were similar to those of transcriptome analysis, demonstrating the reliability of the transcriptome data (Figure 8b).

### 3.8. Relationship between LITCHI029356.m1 Expression and Anthocyanin Accumulation in Fruit Pericarps

**Figure 9:**
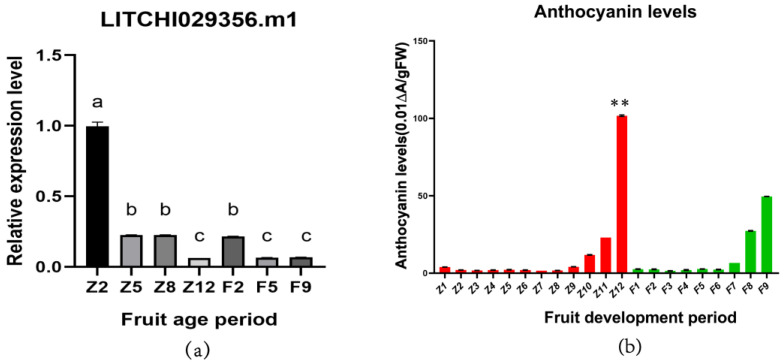
Relationship Between *LITCHI029356.m1* Expression and Anthocyanin Accumulation in Fruit Pericarps. Z8 was significantly higher than Z12 (*p* < 0.01). In the figure, ** indicates that the difference between Z12 and F9 at the mature stage of the two varieties is extremely significant (*p* < 0.01). (**a**) The relative expression levels of the *LITCHI029356.m1* gene in the pericarps of ZNX and FZX lychees at different fruit ages. (**b**) Total anthocyanin content in the pericarps of ZNX and FZX lychees at different fruit ages. Each sample had three biological replicates and three technical replicates. The vertical axis represents the relative expression level of genes. F2 (green peel), F5 (green peel), and harvest maturity period F9 (red peel) represent the pericarp of FZX lychee at the 2nd, 5th, and 9th fruit ages, while Z2 (green peel), Z5 (green peel), Z8 (green peel), and harvest maturity period Z12 (red peel) represent the pericarp of the ZNX lychee at the 2nd, 5th, 8th, and 12th fruit ages, respectively. Data were presented as a mean (±SE) from three independent biological replicates. Different letters indicate significant differences among treatments at *p* < 0.05 level.

The expression of *LITCHI029356.m1* gene in the pericarp of FZX and ZNX varieties at different fruit ages was further studied. When the ZNX fruits began to color (Z8-Z12), the gene expression decreased sharply; however, there was no significant decrease in the fruit coloration stage of FZX(F5-F9). The content of total anthocyanin in the pericarp of the two varieties at each fruit age showed that the content of total anthocyanin in the pericarp of the ZNX lychee was much higher than that of the FZX lychee at maturity, when it showed a sudden increase (Figure 9).

The gene expression data, combined with the changes of anthocyanin content in pericarp during fruit development, showed that anthocyanins in the pericarp of the two varieties gradually accumulated with the decrease in *LITCHI029356.m1* expression in pericarp. This indicates that *LITCHI029356.m1* in lychee plays a negative regulatory role in the accumulation of anthocyanin in the pericarp of ZNX and FZX lychee. At the same time, the difference in the expression of *LITCHI029356.m1* in the two lychee varieties also affected the difference of seed coloring in pericarps of the two varieties. The *LITCHI029356.m1* content in the early development of the ZNX lychee was much higher than that in the FZX lychee at the same period, and the expression of *LITCHI029356.m1* in the mature ZNX late development was significantly decreased, and anthocyanin accumulation was significantly increased. The expression level of *LITCHI029356.m1* in FZX ripening fruit was not significantly decreased, and the content of anthocyanin in ‘Feizixiao’ ripening fruit was much lower than that in ZNX ripening fruit (Figure 9).

**Figure 10:**
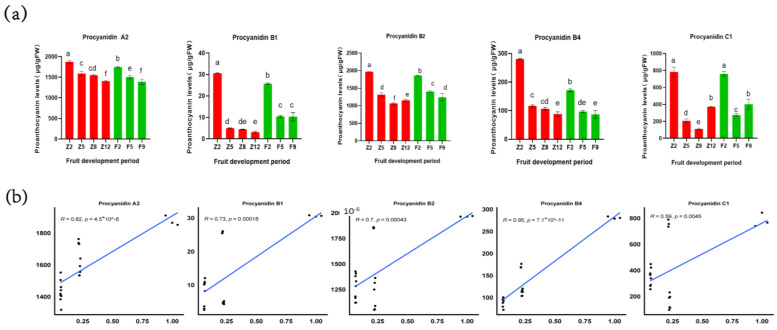
Analysis of proanthocyanidins content: (**a**) the content of proanthocyanidins in the pericarp of the ZNX and FZX lychee at different fruit ages; (**b**) correlation analysis between proanthocyanidins and *LcANR*(*LITCHI029356.m1*). Each sample had three biological replicates and three technical replicates. The vertical axis represents the relative expression level of genes. F2 (green peel), F5 (green peel), and harvest maturity period F9 (red peel) represent the pericarp of FZX lychee at the 2nd, 5th, and 9th fruit ages, while Z2 (green peel), Z5 (green peel), Z8 (green peel), and harvest maturity period Z12 (red peel) represent the pericarp of the ZNX lychee at the 2nd, 5th, 8th, and 12th fruit ages, respectively. Data were presented as a mean (±SE) from three independent biological replicates. Different letters indicate significant differences among treatments at *p* < 0.05 level at *p* < 0.05 level.

Through the determination of proanthocyanidins in the peel and the correlation analysis between proanthocyanidins and *LcANR(LITCHI029356.m1)* gene, we found that the expression level of *LcANR(LITCHI029356.m1)* gene was significantly correlated with the content of proanthocyanidin A2, proanthocyanidin B1, proanthocyanidin B2, and proanthocyanidin B4 in the five proanthocyanidins detected in the peel (r > 0.7). These results indicate that *LcANR(LITCHI029356.m1)* is the dominant gene regulating the generation of these proanthocyanins. With the regulation of *LcANR(LITCHI029356.m1)*, the generation of colorless anthocyanins will lead to a decrease in anthocyanin production, further indicating that the expression of *LcANR(LITCHI029356.m1)* plays a negative regulatory role in anthocyanin production (Figure 10).

### 3.9. Heterologous Expression of LcANR(LITCHI029356.m1) in Tobacco

**Figure 11:**
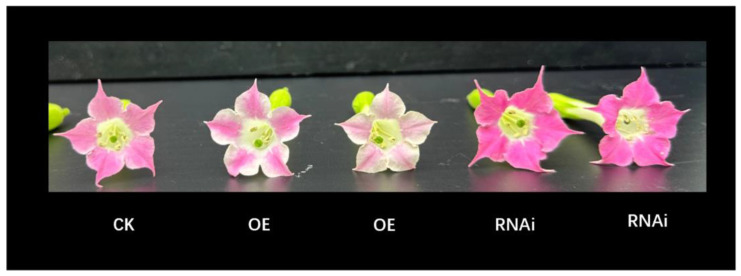
Transgenic tobacco petal phenotype. CK is wild type. OE is the flower of overexpressing plants. RNAi is the flower of silenced *ANR* gene plants.

To explore the role of the lychee *ANR* gene *LITCHI029356.m1* as a potential negative regulator of pigmentation, we overexpressed this gene heterologously in tobacco variety K326, a red-petal cultivar with anthocyanin accumulated in petals. The results showed that the tobacco petal color turned lighter with the expression of *LcANR*(*LITCHI029356.m1*) in tobacco (Figure 10), suggesting down-regulation of anthocyanin accumulation in tobacco flowers with the heterologous expression of *LcANR*(*LITCHI029356.m1*). Furthermore, we suppressed the expression of the orthologous gene in tobacco with RNAi, and the results showed darker petals of the transgenic tobacco plants (Figure 11), indicating that suppression of the gene expression facilitated the accumulation of anthocyanin in tobacco petals. These results accumulatively confirmed the function of *LcANR*(*LITCHI029356.m1*) in negative regulation of anthocyanin biosynthesis in plants.

## 4. Discussion

Anthocyanins and proanthocyanidins are both types of flavonoids, commonly acting as antioxidants in plants. We started our study with the gene identification enabled by the lychee reference genome. As a result, 51 anthocyanidin reductase genes were identified in the lychee genome. The function of the anthocyanidin reductase group was preliminarily predicted through the sequence and phylogenetic analysis of gene family members, and the expression patterns of the family members in different tissues and varieties were analyzed using transcriptome sequencing and RT-qPCR. The expression patterns of lychee *ANR* gene family members vary among tissues, and many low-expression and zero-expression members were identified. For example, *LITCHI011046.m1*, *LITCHI009870.m1*, *LITCHI011049.m1*, *ITCHI004431.m1*, and *LITCHI022853.m1* were not expressed in some tissues. The subcellular targeting results of the *ANR* gene family in lychees is different from the reported *ANR* gene family results in other species, such as tea plant. *ANR* members in tea tree are located in a variety of organelles [28], but *ANR* members in lychee are mostly located in the cytoplasm.

Anthocyanin and proanthocyanidin synthesis share the same substrate and convert to each other, so they compete with each other in the flavonoid metabolic network. Therefore, the study of anthocyanin reductase is very meaningful for the study of lychee pericarp coloring. In this paper, the anthocyanin reductase gene family of lychee was comprehensively identified, and its sequence, phylogeny, and expression characteristics were studied in depth. On the basis of evolutionary and expression analyses, *LcANR*(*LITCHI029356.m1*), which is an *ANR* family member, was identified. *LcANR*(*LITCHI029356.m1*) potentially encodes an enzyme synthesizing proanthocyanidin and acts as a key player in the competition with the anthocyanin accumulation in lychee pericarps. Our work lays the groundwork for the exploration of the lychee *ANR* family, paving the way for an in-depth understanding of the genetic underpinnings that govern the biosynthesis of both anthocyanins and proanthocyanidins.

To investigate the role of the *ANR* genes in lychee (pro)anthocyanin biosynthesis, two varieties with different modes of anthocyanin accumulation were used, and their expression patterns in different developmental stages were characterized through both transcriptome and RT-qPCR. The results show that *LITCHI029356.m1* might play a key role in proanthocyanin biosynthesis. The functioning of this gene seems to lead to competition with anthocyanin accumulation, hence regulating lychee fruit skin coloring. Moreover, we found the transgenic tobacco experiments were consistent with negative regulation of pigmentation via the particular lychee *ANR* gene *LITCHI029356.m1*, probably via anthocyanin biosynthesis. This gene can be used as a potential target for breeding and genetic engineering to customize the pigment composition and distribution in lychee skin to enhance its visual attractiveness.

## 5. Conclusions

In summary, we identified many, possibly all, *ANR* gene family members in the *L. sinensis* genome and investigated their sequence, phylogeny, and expression characteristics in lychee, leading to the identification of a gene potentially acting as a key player in proanthocyanin biosynthesis in lychee. The function of the identified *LcANR* gene was confirmed by both heterologous expression and RNAi. Our work thus serves as a valuable starting point for further exploration into the enzymatic pathways and genetic control mechanisms to regulate the flavonoid accumulation in lychee.

## Figures and Tables

**Table 1:** Top 10 cis-acting elements in the *ANR* promoters in lychee.

No.	Motif Name	Genes Contained	Major Function
1	CAAT-box	50	Promoters and enhancers
2	TATA-box	50	Core promoter element
3	ARE	46	Essential for anaerobic induction
4	Box 4	45	Involved in light response
5	G-box	45	Participate in light reactions
6	ABRE	41	Involved in abscisic acid reaction
7	TCT-motif	29	Light responsive element
8	GT1-motif	28	Light responsive element
9	MBS	24	MYB binding site involved in drought-inducibility
10	TCA-element	20	Involved in salicylic acid responsiveness

## Data Availability

Publicly available datasets were analyzed in this study. This data can be found here: National Center of Biotechnology Information (NCBI) (https://www.ncbi.nlm.nih.gov/, accessed on 30 May 2024) under BioProject PRJNA1117045.

## Supplementary Materials

The following supporting information can be downloaded at: https://www.mdpi.com/article/10.3390/genes15060757/s1, Table S1: Properties of *ANR* family members in litchi; Table S2. Primer information of lychee *ANR* genes for qRT-PCR; Table S3. Primer sequence construction of *LcANR-OE* overexpression vector; Table S4. *LcANR(LITCHI029356.m1)-OE* carrier sequence; Table S5. Primer sequence constructed by *ANR-RNAi* vector; Table S6. *ANR-RNAi* carrier sequence.
