# Supplementary material for: Genome-Wide Identification and Analysis of Anthocyanidin Reductase Gene Family in Lychee (Litchi chinensis Sonn.)
Source: Genes (Basel). 2024 Jun 8;15(6):757. doi: 10.3390/genes15060757 (PMC11202510; doi:10.3390/genes15060757)
Supplement: Supplementary file 1 [file genes-15-00757-s001.zip › S1.pdf]

Table S1. Properties of ANR family members in litchi.

Sequence ID	Number of Amino Acid	Molecular Weight	Theoretical pI	Instability Index	Aliphatic Index	Grand Average of Hydropathicity	Subcellular localization
LITCHI016002.m1	226	24797.35	6.25	25.92	94.07	0.026	Cytoplasmic
LITCHI015995.m1	205	23140.74	5.05	32.6	98	−0.016	Cytoplasmic
LITCHI016572.m1	289	32401.46	5.5	29.14	84.33	−0.192	Cytoplasmic
LITCHI014912.m3	240	27165.94	6.33	47.44	69.54	−0.295	Cytoplasmic
LITCHI025548.m1	241	27009.14	6.76	34.61	99.83	−0.022	Cytoplasmic
LITCHI025531.m1	238	25993.64	6.71	28.64	88.57	0.019	Cytoplasmic
LITCHI027754.m2	235	26255.01	6.18	29.41	94.09	−0.173	Cytoplasmic
LITCHI026246.m1	211	22942.96	5.26	31.55	78.01	−0.22	Cytoplasmic
LITCHI026912.m1	259	28405.22	4.83	34.77	92.59	0.042	Cytoplasmic
LITCHI025537.m1	231	25381.9	6.72	30.3	86.19	−0.044	Cytoplasmic
LITCHI026910.m1	259	28436.32	4.9	34.8	93.36	0.049	Cytoplasmic
LITCHI030943.m1	230	25116.79	5.4	28.57	100.39	0.041	Cytoplasmic
LITCHI002216.m1	243	26774.78	6.26	38.19	97.45	−0.024	Cytoplasmic
LITCHI001132.m1	241	26281.04	5.7	32.72	94.65	−0.025	Cytoplasmic
LITCHI004176.m1	218	24488.15	7.18	27.26	94.31	−0.208	Cytoplasmic
LITCHI003640.m1	240	27207.98	6.15	49.36	71.17	−0.276	Cytoplasmic
LITCHI010262.m1	241	26904.08	5.79	30.61	92.99	−0.014	Cytoplasmic
LITCHI009877.m2	233	25220.2	5.69	31.15	105.41	0.168	Cytoplasmic
LITCHI011049.m1	87	9491.86	5.54	19.56	78.51	−0.038	Cytoplasmic
LITCHI011050.m1	232	25064.36	5.65	29.97	90.34	0.01	Cytoplasmic
LITCHI009873.m1	238	26083.97	6.01	27.96	99.45	−0.034	Cytoplasmic
LITCHI009876.m1	238	26069.02	5.41	38.59	91.72	−0.025	Cytoplasmic
LITCHI009876.m3	190	20709.92	5.58	39.22	86.68	0.043	Cytoplasmic
LITCHI011046.m1	89	9719.04	4.9	23.7	75.62	−0.072	Cytoplasmic
LITCHI010261.m1	241	26960.25	6.26	38.15	86.56	−0.092	Cytoplasmic
LITCHI009870.m1	244	26883.38	8.66	30.9	107.46	0.132	Cytoplasmic
LITCHI011057.m1	218	23394.41	5.63	27.63	91.24	0.008	Cytoplasmic
LITCHI009875.m1	238	26283.32	6.34	29.94	97.86	−0.05	Cytoplasmic
LITCHI029352.m1	244	26268.41	5.78	24.49	103.89	0.127	Cytoplasmic
LITCHI029358.m1	204	21883.13	7.87	21.81	101.81	0.022	Cytoplasmic
LITCHI029356.m1	246	26375.51	5.58	22.14	103.41	0.149	Cytoplasmic
LITCHI021857.m2	263	28868.2	6.38	35.09	100.42	0.029	Cytoplasmic
LITCHI019935.m1	240	25904.72	5.66	24.5	99.5	0.045	Cytoplasmic
LITCHI024534.m1	271	30572.38	6.72	50.04	98.63	−0.07	Cytoplasmic
LITCHI023913.m1	238	25633.42	5.6	25.52	99.12	0.098	Cytoplasmic
LITCHI025184.m1	238	26101.18	5.55	25.43	102.82	0.118	Cytoplasmic
LITCHI004431.m1	252	27493.61	5.68	33.36	97.46	0.06	Cytoplasmic
LITCHI004424.m1	207	22380.83	7.09	22.17	100.29	0.081	Cytoplasmic
LITCHI005521.m1	237	26096.93	6.09	33.21	97.34	−0.014	Cytoplasmic
LITCHI004430.m1	249	27107.15	5.96	32.42	100.68	0.08	Cytoplasmic
LITCHI014994.m1	238	26216.76	6.76	32.82	86.85	−0.125	Periplasmic
LITCHI015992.m1	234	25551.04	8.44	30.66	85.85	−0.104	Periplasmic
LITCHI025943.m1	124	13945.88	8.01	35.45	73.15	−0.327	Periplasmic

LITCHI022853.m1	144	15695.03	9.33	29.39	90.76	0.021	Periplasmic
LITCHI019938.m1	240	25820.65	5.42	23.29	97.04	0.1	Periplasmic
LITCHI019936.m1	240	25776.6	5.42	22.15	97.88	0.107	Periplasmic
LITCHI004425.m1	196	21273.37	7.04	21.13	93.57	0.03	Periplasmic
LITCHI004433.m1	245	26977.19	7.73	29.62	93.88	−0.031	Periplasmic
LITCHI001933.m1	217	23457.07	9.14	39.77	101.06	0.094	Inner Membrane
LITCHI013343.m2	254	27673.69	6.25	32.05	98.98	0.146	Outer Membrane
LITCHI010244.m1	211	23269.55	8.37	38	90.09	−0.214	Outer Membrane
