# Supplementary material for: Genome-Wide Identification and Analysis of Anthocyanidin Reductase Gene Family in Lychee (Litchi chinensis Sonn.)
Source: Genes (Basel). 2024 Jun 8;15(6):757. doi: 10.3390/genes15060757 (PMC11202510; doi:10.3390/genes15060757)
Supplement: Supplementary file 1 [file genes-15-00757-s001.zip › S2.pdf]

Table S2. Primer information of lychee ANR genes for qRT-PCR.

Gene Name	Upstream primer sequence	Downstream primer sequences
LITCHI019936.m1	GCAAGCCAACACAAGAAGCAGAAG	GTAGCCACGAGCCAGAAGAAGC
LITCHI030943.m1	GGGCGGAGGCGAAGGAGAG	ATTGGCGTAAGTCTTGGCTGAGC
LITCHI010261.m1	CCGCTACCTTTGCTCCTCAGAATC	TGTCATCCACGCCTTTGAACTCTG
LITCHI023913.m1	GCCACTGTCCGTGATCCCAATG	CCCATCACACCCATCAACCACAG
LITCHI025548.m1	GTGGCTGGTGATGAGGCTTCTTC	GATGCTCCTGGTAGGTTCGTGATG
LITCHI015992.m1	GCTCTTCGACACCGTGGCATTC	GAGCGTGACGAGACCAGCAATG
LITCHI019935.m1	GCCACTGTCCGTGATCCCAATG	CCCATCACACCCATCAACCACAG
LITCHI015995.m1	CATGTGGCCGGTACTTTTGC	CACTTCACTCCCTTGGCACT
LITCHI025184.m1	ATAACGCCCAGCCCTAAGTG	CAGCCAACGTCTTGGAAAGC
LITCHI029356.m1	TTCCTCAGCAAACGATACCCTCAG	TCTCAGACGAAAGGGACAACTTGG
