# Supplementary material for: Genome-Wide Identification and Analysis of Anthocyanidin Reductase Gene Family in Lychee (Litchi chinensis Sonn.)
Source: Genes (Basel). 2024 Jun 8;15(6):757. doi: 10.3390/genes15060757 (PMC11202510; doi:10.3390/genes15060757)
Supplement: Supplementary file 1 [file genes-15-00757-s001.zip › S3.pdf]

Table S3. Primer sequence construction of LcANR-OE overexpression vector.

Gene Name	Upstream primer sequence	Downstream primer sequences
ANR-OE	ACTAGGGTCTCGCACCATGGCCAGCGAGTTCACCGG	CTAGGGTCTCTCGCCCTTAAGCAGCCCCCTAGTCTTCAAGTACTCC
35S-Egfp	CACGGGGGACTCTTGCCACC	GACACGCTGAACTTGTGG
