# Supplementary material for: Genome-Wide Identification and Analysis of Anthocyanidin Reductase Gene Family in Lychee (Litchi chinensis Sonn.)
Source: Genes (Basel). 2024 Jun 8;15(6):757. doi: 10.3390/genes15060757 (PMC11202510; doi:10.3390/genes15060757)
Supplement: Supplementary file 1 [file genes-15-00757-s001.zip › S4.pdf]

Table S4. ***LcANR(LITCHI029356.m1)-OE*** **carrier sequence**

taatgtgagttagctcactcattaggcaccccaggctttacactttatgcttccggctcgtatgttgtgtggaattgtgagcggataa
caatttcacacaggaaacagctatgacatgattacgaattcCATGGAGTCAAAGATTCAAATAGAGGACCTAAC
AGAACTCGCCGTAAAGACTGGCGAACAGTTCATACAGAGTCTCTTACGACTCAATGACAAGAA
GAAAATCTTCGTCAACATGGTGGAGCACGACACACTTGTCTACTCCAAAAATATCAAAGATACA
GTCTCAGAAGACCAAAGGGCAATTGAGACTTTTCAACAAAGGGTAATATCCGGAAACCTCCTCG
GATTCCATTGCCCAGCTATCTGTCACTTTATTGTGAAGATAGTGGAAAAGGAAGGTGGCTCCTAC
AAATGCCATCATTGCGATAAAGGAAAGGCCATCGTTGAAGATGCCTCTGCCGACAGTGGTCCC
AAAGATGGACCCCCACCCACGAGGAGCATCGTGGAAAAAGAAGACGTTCCAACCACGTCTTCA
AAGCAAGTGGATTGATGTGATATCTCCACTGACGTAAGGGATGACGCACAATCCCACTATCCTT
CGCAAGACCCTTCCTCTATATAAGGAAGTTCATTTCATTTGGAGAGAACACGGGGGACTCTTGC
CACCATGGCCAGCGAGTTCACCGGAAAAAAGACCGCATGTGTAGTTGGTGGCACCGGATATGT
GGCCTCCTTGTTGATCAAATTGTTGCTTCAAAAGGGCTATGTTGTTCACACTACAGTCAGAGACC
CAGCGAACCGGAAAAAGATCTCCCACCTAATAGCACTACAAGAATTAGGTGAGCTGAAAATTTT
TGGAGCAGATTTGACCGATGAAGGGAGTTTTGATGCCCCTGTAGAGGGTTGTGACATTGTCTTC
CATGTTGCAACACCAGTCAATTTTGCTTCTGAAGATCCAGAGAATGACATGATCAAGCCAGCTA
TTAAAGGAGTGTTGAATGTTTTGAAAGCCTGTGCAAAGGCAAAATCAGTTAAACGAGTCGTAGT
GACATCTTCAGCTGCTGCCGTGACAATCAATAAACTTAATGGAACAGGTTTGGTCATGGATGAG
AAAAACTGGAGTGATGTTGAGTTTTTGAGCTCTGAGAAACCACCAACTTGGGGGTACCCTGTAT
CCAAGACCTTGGCTGAAAAGGAAGCTTGGAAATTTGCTCAGGAGAACAACATTGATCTAGTTAC
TGTCATCCCTACTCTCATGACCGGTCCTGCTCTCACGCCAGATATTCCAAGCTCCATTGGCCTCG
CCACGTCCTTGATTACAGGGAATGAGTTTCTCATAAATGCTATAAAAGGCATGCAAATGCTGTCA
GGTTCATTGTCCATTACACATGTGGAGGATGTTTGCAGAGCCCATATTTTTGTGGCTGAGAAAGA
ATCTGCTTCAGGTCGATACATTTGTGCTGCTTTCAACACCAGTGTTCCTGAGGCTGCTAAGTTCC
TCAGCAAACGATACCCTCAGTACAAAATTCCAACCGATTTTGGAGACTTTCCCACCAAGGCCAA
GTTGTCCCTTTCGTCTGAGAAGCTTATTAGTGAAGGATTCAGCTTCAAGTATGGGCTTGAAGAAA
CCTATGATCAAACTGTGGAGTACTTGAAGACTAGGGGGCTGCTTAAGGGCGGCGGCGGCTCCG
GCGGCGGCGGCTCCATGGTGAGCAAGGGCGAGGAGCTGTTCACCGGGGTGGTGCCCATCCTG
GTCGAGCTGGACGGCGACGTAAACGGCCACAAGTTCAGCGTGTCCGGCGAGGGCGAGGGCGA
TGCCACCTACGGCAAGCTGACCCTGAAGTTCATCTGCACCACCGGCAAGCTGCCCGTGCCCTG
GCCCACCCTCGTGACCACCCTGACCTACGGCGTGCAGTGCTTCAGCCGCTACCCCGACCACAT
GAAGCAGCACGACTTCTTCAAGTCCGCCATGCCCGAAGGCTACGTCCAGGAGCGCACCATCTT
CTTCAAGGACGACGGCAACTACAAGACCCGCGCCGAGGTGAAGTTCGAGGGCGACACCCTGG
TGAACCGCATCGAGCTGAAGGGCATCGACTTCAAGGAGGACGGCAACATCCTGGGGCACAAG
CTGGAGTACAACTACAACAGCCACAACGTCTATATCATGGCCGACAAGCAGAAGAACGGCATC
AAGGTGAACTTCAAGATCCGCCACAACATCGAGGACGGCAGCGTGCAGCTCGCCGACCACTAC
CAGCAGAACACCCCCATCGGCGACGGCCCCGTGCTGCTGCCCGACAACCACTACCTGAGCACC
CAGTCCGCCCTGAGCAAAGACCCCAACGAGAAGCGCGATCACATGGTCCTGCTGGAGTTCGTG
ACCGCCGCCGGGATCACTCTCGGCATGGACGAGCTGTACAAGTGACGGTGATCCTCCCGATCG
TTCAAACATTTGGCAATAAAGTTTCTTAAGATTGAATCCTGTTGCCGGTCTTGCGATGATTATCAT
ATAATTTCTGTTGAATTACGTTAAGCATGTAATAATTAACATGTAATGCATGACGTTATTTATGAG
GTGGGTTTTTATGATTAGAGTCCCGCAATTATACATTTAATACGCGATAGAAAACAAAATATAGC
GCGCAAACTAGGATAAATTATCGCGCGCGGTGTCATCTATGTTACTAGATCGGGAGCACCGGTA
AGGCGCGCCGTAGTGaagcttggcactggccgtcgttttacaacgtcgtgactgggaaaaccctggcgttacccaactta

atcgccttgcagcacatccccctttcgccagctggcgtaatagcgaagaggcccgcaccgatcgcccttcccaacagttgcgca
gcctgaatggcgaatgctagagcagcttgagcttggatcagattgtcgtttcccgccttcagtttaaactatcagtgtttgacagg
atatattggcgggtaaacctaagagaaaagagcgtttattagaataacggatatttaaaagggcgtgaaaaggtttatccgttcg
tccatttgtatgtgcatgccaaccacagggttcccctcgggatcaaagtactttgatccaacccctccgctgctatagtgcagtcgg
cttctgacgttcagtgcagccgtcttctgaaaacgacatgtcgcacaagtcctaagttacgcgacaggctgccgccctgccctttc
ctggcgttttcttgtcgcgtgttttagtcgcataaagtagaatacttgcgactagaaccggagacattacgccatgaacaagagc
gccgccgctggcctgctgggctatgcccgcgtcagcaccgacgaccaggacttgaccaaccaacgggccgaactgcacgcg
gccggctgcaccaagctgttttccgagaagatcaccggcaccaggcgcgaccgcccggagctggccaggatgcttgaccacc
tacgccctggcgacgttgtgacagtgaccaggctagaccgcctggcccgcagcacccgcgacctactggacattgccgagcg
catccaggaggccggcgcgggcctgcgtagcctggcagagccgtgggccgacaccaccacgccggccggccgcatggtgt
tgaccgtgttcgccggcattgccgagttcgagcgttccctaatcatcgaccgcacccggagcgggcgcgaggccgccaaggc
ccgaggcgtgaagtttggcccccgccctaccctcaccccggcacagatcgcgcacgcccgcgagctgatcgaccaggaaggc
cgcaccgtgaaagaggcggctgcactgcttggcgtgcatcgctcgaccctgtaccgcgcacttgagcgcagcgaggaagtga
cgcccaccgaggccaggcggcgcggtgccttccgtgaggacgcattgaccgaggccgacgccctggcggccgccgagaat
gaacgccaagaggaacaagcatgaaaccgcaccaggacggccaggacgaaccgtttttcattaccgaagagatcgaggcg
gagatgatcgcggccgggtacgtgttcgagccgcccgcgcacgtctcaaccgtgcggctgcatgaaatcctggccggtttgtc
tgatgccaagctggcggcctggccggccagcttggccgctgaagaaaccgagcgccgccgtctaaaaaggtgatgtgtattt
gagtaaaacagcttgcgtcatgcggtcgctgcgtatatgatgcgatgagtaaataaacaaatacgcaaggggaacgcatgaa
ggttatcgctgtacttaaccagaaaggcgggtcaggcaagacgaccatcgcaacccatctagcccgcgccctgcaactcgccg
gggccgatgttctgttagtcgattccgatccccagggcagtgcccgcgattgggcggccgtgcgggaagatcaaccgctaacc
gttgtcggcatcgaccgcccgacgattgaccgcgacgtgaaggccatcggccggcgcgacttcgtagtgatcgacggagcg
ccccaggcggcggacttggctgtgtccgcgatcaaggcagccgacttcgtgctgattccggtgcagccaagcccttacgacat
atgggccaccgccgacctggtggagctggttaagcagcgcattgaggtcacggatggaaggctacaagcggcctttgtcgtg
tcgcgggcgatcaaaggcacgcgcatcggcggtgaggttgccgaggcgctggccgggtacgagctgcccattcttgagtcc
cgtatcacgcagcgcgtgagctacccaggcactgccgccgccggcacaaccgttcttgaatcagaacccgagggcgacgctg
cccgcgaggtccaggcgctggccgctgaaattaaatcaaaactcatttgagttaatgaggtaaagagaaaatgagcaaaagc
acaaacacgctaagtgccggccgtccgagcgcacgcagcagcaaggctgcaacgttggccagcctggcagacacgccagc
catgaagcgggtcaactttcagttgccggcggaggatcacaccaagctgaagatgtacgcggtacgccaaggcaagaccatt
accgagctgctatctgaatacatcgcgcagctaccagagtaaatgagcaaatgaataaatgagtagatgaattttagcggctaa
aggaggcggcatggaaaatcaagaacaaccaggcaccgacgccgtggaatgccccatgtgtggaggaacgggcggttgg
ccaggcgtaagcggctgggttgtctgccggccctgcaatggcactggaacccccaagcccgaggaatcggcgtgacggtcg
caaaccatccggcccggtacaaatcggcgcggcgctgggtgatgacctggtggagaagttgaaggccgcgcaggccgccc
agcggcaacgcatcgaggcagaagcacgccccggtgaatcgtggcaagcggccgctgatcgaatccgcaaagaatcccgg
caaccgccggcagccggtgcgccgtcgattaggaagccgcccaagggcgacgagcaaccagatttttcgttccgatgctcta
tgacgtgggcacccgcgatagtcgcagcatcatggacgtggccgttttccgtctgtcgaagcgtgaccgacgagctggcgag
gtgatccgctacgagcttccagacgggcacgtagaggtttccgcagggccggccggcatggccagtgtgtgggattacgacc
tggtactgatggcggtttcccatctaaccgaatccatgaaccgataccgggaagggaagggagacaagcccggccgcgtgtt
ccgtccacacgttgcggacgtactcaagttctgccggcgagccgatggcggaaagcagaaagacgacctggtagaaacctg
cattcggttaaacaccacgcacgttgccatgcagcgtacgaagaaggccaagaacggccgcctggtgacggtatccgagggt
gaagccttgattagccgctacaagatcgtaaagagcgaaaccgggcggccggagtacatcgagatcgagctagctgattgga
tgtaccgcgagatcacagaaggcaagaacccggacgtgctgacggttcaccccgattactttttgatcgatcccggcatcggcc
gttttctctaccgcctggcacgccgcgccgcaggcaaggcagaagccagatggttgttcaagacgatctacgaacgcagtggc
agcgccggagagttcaagaagttctgtttcaccgtgcgcaagctgatcgggtcaaatgacctgccggagtacgatttgaagga
ggaggcggggcaggctggcccgatcctagtcatgcgctaccgcaacctgatcgagggcgaagcatccgccggttcctaatgt

acggagcagatgctagggcaaattgccctagcaggggaaaaaggtcgaaaaggtTtctttcctgtggatagcacgtacattg
ggaacccaaagccgtacattgggaaccggaacccgtacattgggaacccaaagccgtacattgggaaccggtcacacatgta
agtgactgatataaaagagaaaaaaggcgatttttccgcctaaaactctttaaaacttattaaaactcttaaaacccgcctggcctg
tgcataactgtctggccagcgcacagccgaagagctgcaaaaagcgcctacccttcggtcgctgcgctccctacgccccgccg
cttcgcgtcggcctatcgcggccgctggccgctcaaaaatggctggcctacggccaggcaatctaccagggcgcggacaagc
cgcgccgtcgccactcgaccgccggcgcccacatcaaggcaccctgcctcgcgcgtttcggtgatgacggtgaaaacctctga
cacatgcagctcccggagacggtcacagcttgtctgtaagcggatgccgggagcagacaagcccgtcagggcgcgtcagcg
ggtgttggcgggtgtcggggcgcagccatgacccagtcacgtagcgatagcggagtgtatactggcttaactatgcggcatc
agagcagattgtactgagagtgcaccatatgcggtgtgaaataccgcacagatgcgtaaggagaaaataccgcatcaggcgc
tcttccgcttcctcgctcactgactcgctgcgctcggtcgttcggctgcggcgagcggtatcagctcactcaaaggcggtaatac
ggttatccacagaatcaggggataacgcaggaaagaacatgtgagcaaaaggccagcaaaaggccaggaaccgtaaaaa
ggccgcgttgctggcgtttttccataggctccgcccccctgacgagcatcacaaaaatcgacgctcaagtcagaggtggcgaa
acccgacaggactataaagataccaggcgtttccccctggaagctccctcgtgcgctctcctgttccgaccctgccgcttaccgg
atacctgtccgcctttctcccttcgggaagcgtggcgctttctcatagctcacgctgtaggtatctcagttcggtgtaggtcgttcgc
tccaagctgggctgtgtgcacgaaccccccgttcagcccgaccgctgcgccttatccggtaactatcgtcttgagtccaacccgg
taagacacgacttatcgccactggcagcagccactggtaacaggattagcagagcgaggtatgtaggcggtgctacagagtt
cttgaagtggtggcctaactacggctacactagaaggacagtatttggtatctgcgctctgctgaagccagttaccttcggaaaa
agagttggtagctcttgatccggcaaacaaaccaccgctggtagcggtggtttttttgtttgcaagcagcagattacgcgcagaa
aaaaaggatctcaagaagatcctttgatcttttctacggggtctgacgctcagtggaacgaaaactcacgttaagggattttggtc
atgcattctaggtactaaaacaattcatccagtaaaatataatattttattttctcccaatcaggcttgatccccagtaagtcaaaaaa
tagctcgacatactgttcttccccgatatcctccctgatcgaccggacgcagaaggcaatgtcataccacttgtccgccctgccgct
tctcccaagatcaataaagccacttactttgccatctttcacaaagatgttgctgtctcccaggtcgccgtgggaaaagacaagttc
ctcttcgggcttttccgtctttaaaaaatcatacagctcgcgcggatctttaaatggagtgtcttcttcccagttttcgcaatccacatc
ggccagatcgttattcagtaagtaatccaattcggctaagcggctgtctaagctattcgtatagggacaatccgatatgtcgatgg
agtgaaagagcctgatgcactccgcatacagctcgataatcttttcagggctttgttcatcttcatactcttccgagcaaaggacgc
catcggcctcactcatgagcagattgctccagccatcatgccgttcaaagtgcaggacctttggaacaggcagctttccttccagc
catagcatcatgtccttttcccgttccacatcataggtggtccctttataccggctgtccgtcatttttaaatataggttttcattttctccc
accagcttatataccttagcaggagacattccttccgtatcttttacgcagcggtatttttcgatcagttttttcaattccggtgatattc
tcattttagccatttattatttccttcctcttttctacagtatttaaagataccccaagaagctaattataacaagacgaactccaattcac
tgttccttgcattctaaaaccttaaataccagaaaacagctttttcaaagttgttttcaaagttggcgtataacatagtatcgacgga
gccgattttgaaaccgcggtgatcacaggcagcaacgctctgtcatcgttacaatcaacatgctaccctccgcgagatcatccgt
gtttcaaacccggcagcttagttgccgttcttccgaatagcatcggtaacatgagcaaagtctgccgccttacaacggctctcccg
ctgacgccgtcccggactgatgggctgcctgtatcgagtggtgattttgtgccgagctgccggtcggggagctgttggctggct
ggtggcaggatatattgtggtgtaaacaaattgacgcttagacaacttaataacacattgcggacgtttttaatgtactgaattaac
gccgaattaattcgggggatctggattttagtactggattttggttttaggaattagaaattttattgatagaagtattttacaaatac
aaatacatactaagggtttcttatatgctcaacacatgagcgaaaccctataggaaccctaattcccttatctgggaactactcaca
cattattatggagaaactcgagcttgtcgatcgacagatccggtcggcatctactctatttctttgccctcggacgagtgctgggg
cgtcggtttccactatcggcgagtacttctacacagccatcggtccagacggccgcgcttctgcgggcgatttgtgtacgcccga
cagtcccggctccggatcggacgattgcgtcgcatcgaccctgcgcccaagctgcatcatcgaaattgccgtcaaccaagctct
gatagagttggtcaagaccaatgcggagcatatacgcccggagtcgtggcgatcctgcaagctccggatgcctccgctcgaa
gtagcgcgtctgctgctccatacaagccaaccacggcctccagaagaagatgttggcgacctcgtattgggaatccccgaacat
cgcctcgctccagtcaatgaccgctgttatgcggccattgtccgtcaggacattgttggagccgaaatccgcgtgcacgaggtg
ccggacttcggggcagtcctcggcccaaagcatcagctcatcgagagcctgcgcgacggacgcactgacggtgtcgtccatc
acagtttgccagtgatacacatggggatcagcaatcgcgcatatgaaatcacgccatgtagtgtattgaccgattccttgcggtc

cgaatgggccgaacccgctcgtctggctaagatcggccgcagcgatcgcatccatagcctccgcgaccggttgtagaacagc
gggcagttcggtttcaggcaggtcttgcaacgtgacaccctgtgcacggcgggagatgcaataggtcaggctctcgctaaact
ccccaatgtcaagcacttccggaatcgggagcgcggccgatgcaaagtgccgataaacataacgatctttgtagaaaccatcg
gcgcagctatttacccgcaggacatatccacgccctcctacatcgaagctgaaagcacgagattcttcgccctccgagagctgca
tcaggtcggagacgctgtcgaacttttcgatcagaaacttctcgacagacgtcgcggtgagttcaggctttttcatatctcattgcc
ccccgggatctgcgaaagctcgagagagatagatttgtagagagagactggtgatttcagcgtgtcctctccaaatgaaatga
acttccttatatagaggaaggtcttgcgaaggatagtgggattgtgcgtcatcccttacgtcagtggagatatcacatcaatccac
ttgctttgaagacgtggttggaacgtcttctttttccacgatgctcctcgtgggtgggggtccatctttgggaccactgtcggcaga
ggcatcttgaacgatagcctttcctttatcgcaatgatggcatttgtaggtgccaccttccttttctactgtccttttgatgaagtgaca
gatagctgggcaatggaatccgaggaggtttcccgatattaccctttgttgaaaagtctcaatagccctttggtcttctgagactgt
atctttgatattcttggagtagacgagagtgtcgtgctccaccatgttatcacatcaatccacttgctttgaagacgtggttggaac
gtcttctttttccacgatgctcctcgtgggtgggggtccatctttgggaccactgtcggcagaggcatcttgaacgatagcctttcc
tttatcgcaatgatggcatttgtaggtgccaccttccttttctactgtccttttgatgaagtgacagatagctgggcaatggaatccg
aggaggtttcccgatattaccctttgttgaaaagtctcaatagccctttggtcttctgagactgtatctttgatattcttggagtagac
gagagtgtcgtgctccaccatgttggcaagctgctctagccaatacgcaaaccgcctctccccgcgcgttggccgattcattaat
gcagctggcacgacaggtttcccgactggaaagcgggcagtgagcgcaacgcaat

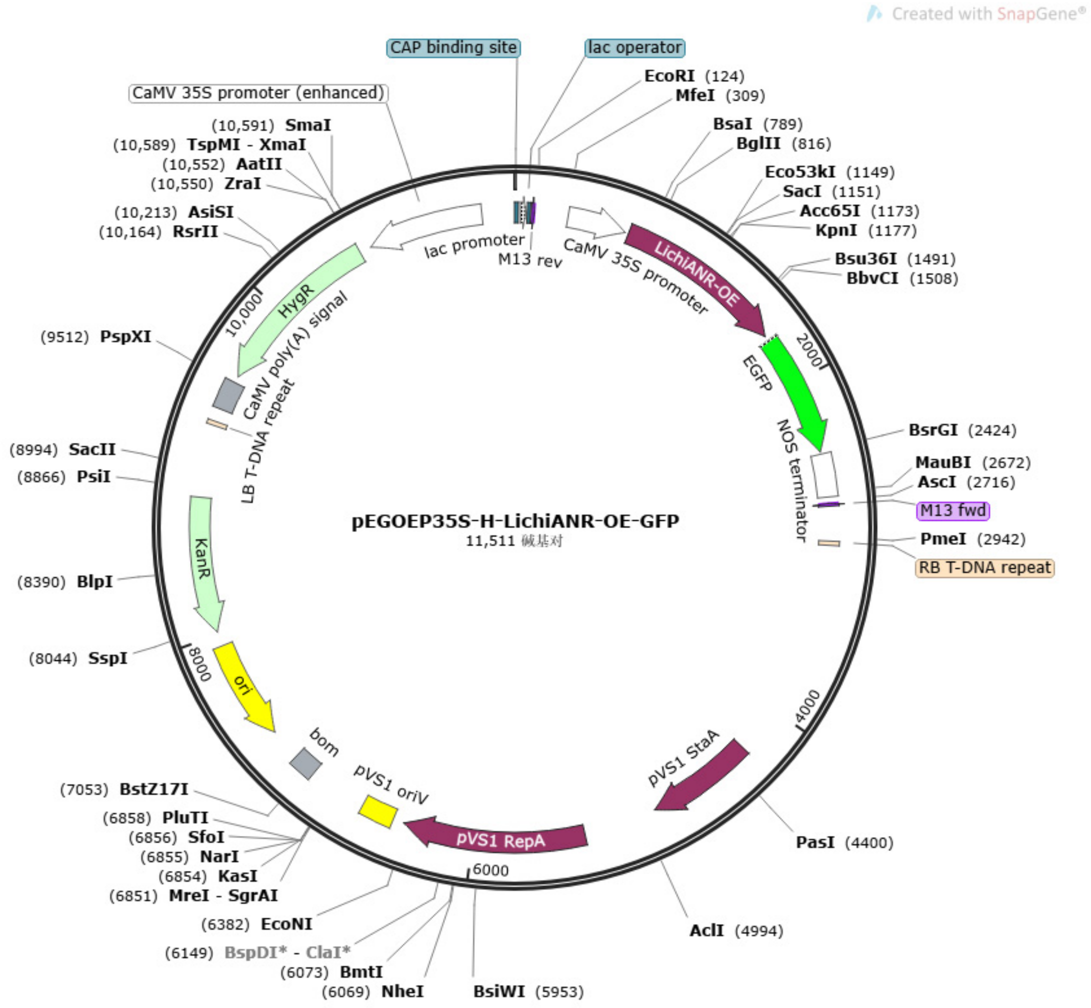

LcANR(LITCHI029356.m1)-OE carrier map
