# Supplementary material for: Genome-Wide Identification and Analysis of Anthocyanidin Reductase Gene Family in Lychee (Litchi chinensis Sonn.)
Source: Genes (Basel). 2024 Jun 8;15(6):757. doi: 10.3390/genes15060757 (PMC11202510; doi:10.3390/genes15060757)
Supplement: Supplementary file 1 [file genes-15-00757-s001.zip › S6.pdf]

Table S6. *ANR-RNAi* carrier sequence

taatgtgagttagctcactcattaggcaccccaggctttacactttatgcttccggctcgtatgttgtgtggaattgtgagcggataa
caatttcacacaggaaacagctatgacatgattacgaattcCATGGAGTCAAAGATTCAAATAGAGGACCTAAC
AGAACTCGCCGTAAAGACTGGCGAACAGTTCATACAGAGTCTCTTACGACTCAATGACAAGAA
GAAAATCTTCGTCAACATGGTGGAGCACGACACACTTGTCTACTCCAAAAATATCAAAGATACA
GTCTCAGAAGACCAAAGGGCAATTGAGACTTTTCAACAAAGGGTAATATCCGGAAACCTCCTCG
GATTCCATTGCCCAGCTATCTGTCACTTTATTGTGAAGATAGTGGAAAAGGAAGGTGGCTCCTAC
AAATGCCATCATTGCGATAAAGGAAAGGCCATCGTTGAAGATGCCTCTGCCGACAGTGGTCCC
AAAGATGGACCCCCACCCACGAGGAGCATCGTGGAAAAAGAAGACGTTCCAACCACGTCTTCA
AAGCAAGTGGATTGATGTGATATCTCCACTGACGTAAGGGATGACGCACAATCCCACTATCCTT
CGCAAGACCCTTCCTCTATATAAGGAAGTTCATTTCATTTGGAGAGAACACGGGGGACTCTTGC
CACCATATTTTTGTTGCTGAGAAAGAATCAGCTTCCGGACGATACATTTGTTCTGCCATCAACAC
CAGTGTTCCGGAGCTAGCAAATTTCTTGAAGAAAAGATATCCAACTTCGGATGTTCCTACAGATT
TCGGGGATTTCCCCTCCAAGGCCAAGTTGATCATCTCGTCAGAAAAGCTCATCAAAGAGGGATT
CAATTTTAAGTATGGGATTGAAGAGATTTATGATCAATGTCTTGCTTGTTTTAAAGATAAGGGGTT
ACTAAAGAACTGAATAGTTCAGTTAATATTGTCTGCAGGTAAATTTCTAGTTTTTCTCCTTCATTTT
CTTGGTTAGGACCCTTTTCTCTTTTTATTTTTTTGAGCTTTGATCTTTCTTTAAACTGATCTATTTTTT
AATTGATTGGTTATGGTGTAAATATTACATAGCTTTAACTGATAATCTGATTACTTTATTTCGTGTG
TCTATGATGATGATGATAGTTACAGAAGCTTACAATATTAACTGAACTATTCAGTTCTTTAGTAAC
CCCTTATCTTTAAAACAAGCAAGACATTGATCATAAATCTCTTCAATCCCATACTTAAAATTGAAT
CCCTCTTTGATGAGCTTTTCTGACGAGATGATCAACTTGGCCTTGGAGGGGAAATCCCCGAAAT
CTGTAGGAACATCCGAAGTTGGATATCTTTTCTTCAAGAAATTTGCTAGCTCCGGAACACTGGTG
TTGATGGCAGAACAAATGTATCGTCCGGAAGCTGATTCTTTCTCAGCAACAAAAATATGGCGGC
GGCGGCTCCGGCGGCGGCGGCTCCATGGTGAGCAAGGGCGAGGAGCTGTTCACCGGGGTGGT
GCCCATCCTGGTCGAGCTGGACGGCGACGTAAACGGCCACAAGTTCAGCGTGTCCGGCGAGG
GCGAGGGCGATGCCACCTACGGCAAGCTGACCCTGAAGTTCATCTGCACCACCGGCAAGCTGC
CCGTGCCCTGGCCCACCCTCGTGACCACCCTGACCTACGGCGTGCAGTGCTTCAGCCGCTACCC
CGACCACATGAAGCAGCACGACTTCTTCAAGTCCGCCATGCCCGAAGGCTACGTCCAGGAGCG
CACCATCTTCTTCAAGGACGACGGCAACTACAAGACCCGCGCCGAGGTGAAGTTCGAGGGCGA
CACCCTGGTGAACCGCATCGAGCTGAAGGGCATCGACTTCAAGGAGGACGGCAACATCCTGGG
GCACAAGCTGGAGTACAACTACAACAGCCACAACGTCTATATCATGGCCGACAAGCAGAAGAA
CGGCATCAAGGTGAACTTCAAGATCCGCCACAACATCGAGGACGGCAGCGTGCAGCTCGCCGA
CCACTACCAGCAGAACACCCCCATCGGCGACGGCCCCGTGCTGCTGCCCGACAACCACTACCT
GAGCACCCAGTCCGCCCTGAGCAAAGACCCCAACGAGAAGCGCGATCACATGGTCCTGCTGG
AGTTCGTGACCGCCGCCGGGATCACTCTCGGCATGGACGAGCTGTACAAGTGACGGTGATCCT
CCCGATCGTTCAAACATTTGGCAATAAAGTTTCTTAAGATTGAATCCTGTTGCCGGTCTTGCGAT
GATTATCATATAATTTCTGTTGAATTACGTTAAGCATGTAATAATTAACATGTAATGCATGACGTT
ATTTATGAGGTGGGTTTTTATGATTAGAGTCCCGCAATTATACATTTAATACGCGATAGAAAACA
AAATATAGCGCGCAAACTAGGATAAATTATCGCGCGCGGTGTCATCTATGTTACTAGATCGGGA
GCACCGGTAAGGCGCGCCGTAGTGaagcttggcactggccgtcgttttacaacgtcgtgactgggaaaaccctggc
gttacccaacttaatcgccttgcagcacatccccctttcgccagctggcgtaatagcgaagaggcccgcaccgatcgcccttccc
aacagttgcgcagcctgaatggcgaatgctagagcagcttgagcttggatcagattgtcgtttcccgccttcagtttaaactatca
gtgtttgacaggatatattggcgggtaaacctaagagaaaagagcgtttattagaataacggatatttaaaagggcgtgaaaa
ggtttatccgttcgtccatttgtatgtgcatgccaaccacagggttcccctcgggatcaaagtactttgatccaacccctccgctgct

atagtgcagtcggcttctgacgttcagtgcagccgtcttctgaaaacgacatgtcgcacaagtcctaagttacgcgacaggctgc
cgccctgcccttttcctggcgttttcttgtcgcgtgttttagtcgcataaagtagaatacttgcgactagaaccggagacattacgcc
atgaacaagagcgccgccgctggcctgctgggctatgcccgcgtcagcaccgacgaccaggacttgaccaaccaacgggcc
gaactgcacgcggccggctgcaccaagctgttttccgagaagatcaccggcaccaggcgcgaccgcccggagctggccagg
atgcttgaccacctacgccctggcgacgttgtgacagtgaccaggctagaccgcctggcccgcagcacccgcgacctactgga
cattgccgagcgcatccaggaggccggcgcgggcctgcgtagcctggcagagccgtgggccgacaccaccacgccggccg
gccgcatggtgttgaccgtgttcgccggcattgccgagttcgagcgttccctaatcatcgaccgcacccggagcgggcgcgag
gccgccaaggcccgaggcgtgaagtttggcccccgccctaccctcaccccggcacagatcgcgcacgcccgcgagctgatcg
accaggaaggccgcaccgtgaaagaggcggctgcactgcttggcgtgcatcgctcgaccctgtaccgcgcacttgagcgca
gcgaggaagtgacgcccaccgaggccaggcggcgcggtgccttccgtgaggacgcattgaccgaggccgacgccctggc
ggccgccgagaatgaacgccaagaggaacaagcatgaaaccgcaccaggacggccaggacgaaccgtttttcattaccgaa
gagatcgaggcggagatgatcgcggccgggtacgtgttcgagccgcccgcgcacgtctcaaccgtgcggctgcatgaaatc
ctggccggtttgtctgatgccaagctggcggcctggccggccagcttggccgctgaagaaaccgagcgccgccgtctaaaaa
ggtgatgtgtatttgagtaaaacagcttgcgtcatgcggtcgctgcgtatatgatgcgatgagtaaataaacaaatacgcaagg
ggaacgcatgaaggttatcgctgtacttaaccagaaaggcgggtcaggcaagacgaccatcgcaacccatctagcccgcgcc
ctgcaactcgccggggccgatgttctgttagtcgattccgatccccagggcagtgcccgcgattgggcggccgtgcgggaag
atcaaccgctaaccgttgtcggcatcgaccgcccgacgattgaccgcgacgtgaaggccatcggccggcgcgacttcgtagtg
atcgacggagcgccccaggcggcggacttggctgtgtccgcgatcaaggcagccgacttcgtgctgattccggtgcagccaa
gcccttacgacatatgggccaccgccgacctggtggagctggttaagcagcgcattgaggtcacggatggaaggctacaagc
ggcctttgtcgtgtcgcgggcgatcaaaggcacgcgcatcggcggtgaggttgccgaggcgctggccgggtacgagctgcc
cattcttgagtcccgtatcacgcagcgcgtgagctacccaggcactgccgccgccggcacaaccgttcttgaatcagaacccga
gggcgacgctgcccgcgaggtccaggcgctggccgctgaaattaaatcaaaactcatttgagttaatgaggtaaagagaaaa
tgagcaaaagcacaaacacgctaagtgccggccgtccgagcgcacgcagcagcaaggctgcaacgttggccagcctggca
gacacgccagccatgaagcgggtcaactttcagttgccggcggaggatcacaccaagctgaagatgtacgcggtacgccaa
ggcaagaccattaccgagctgctatctgaatacatcgcgcagctaccagagtaaatgagcaaatgaataaatgagtagatgaa
ttttagcggctaaaggaggcggcatggaaaatcaagaacaaccaggcaccgacgccgtggaatgccccatgtgtggaggaa
cgggcggttggccaggcgtaagcggctgggttgtctgccggccctgcaatggcactggaacccccaagcccgaggaatcgg
cgtgacggtcgcaaaccatccggcccggtacaaatcggcgcggcgctgggtgatgacctggtggagaagttgaaggccgc
gcaggccgcccagcggcaacgcatcgaggcagaagcacgccccggtgaatcgtggcaagcggccgctgatcgaatccgca
aagaatcccggcaaccgccggcagccggtgcgccgtcgattaggaagccgcccaagggcgacgagcaaccagatttttcgt
tccgatgctctatgacgtgggcacccgcgatagtcgcagcatcatggacgtggccgttttccgtctgtcgaagcgtgaccgacg
agctggcgaggtgatccgctacgagcttccagacgggcacgtagaggtttccgcagggccggccggcatggccagtgtgtg
ggattacgacctggtactgatggcggtttcccatctaaccgaatccatgaaccgataccgggaagggaagggagacaagccc
ggccgcgtgttccgtccacacgttgcggacgtactcaagttctgccggcgagccgatggcggaaagcagaaagacgacctg
gtagaaacctgcattcggttaaacaccacgcacgttgccatgcagcgtacgaagaaggccaagaacggccgcctggtgacgg
tatccgagggtgaagccttgattagccgctacaagatcgtaaagagcgaaaccgggcggccggagtacatcgagatcgagct
agctgattggatgtaccgcgagatcacagaaggcaagaacccggacgtgctgacggttcaccccgattactttttgatcgatcc
cggcatcggccgttttctctaccgcctggcacgccgcgccgcaggcaaggcagaagccagatggttgttcaagacgatctacg
aacgcagtggcagcgccggagagttcaagaagttctgtttcaccgtgcgcaagctgatcgggtcaaatgacctgccggagtac
gatttgaaggaggaggcggggcaggctggcccgatcctagtcatgcgctaccgcaacctgatcgagggcgaagcatccgcc
ggttcctaatgtacggagcagatgctagggcaaattgccctagcaggggaaaaaggtcgaaaaggtTtctttcctgtggatag
cacgtacattgggaacccaaagccgtacattgggaaccggaacccgtacattgggaacccaaagccgtacattgggaaccgg
tcacacatgtaagtgactgatataaaagagaaaaaaggcgatttttccgcctaaaactctttaaaacttattaaaactcttaaaacc
cgcctggcctgtgcataactgtctggccagcgcacagccgaagagctgcaaaaagcgcctacccttcggtcgctgcgctcccta

cgccccgccgcttcgcgtcggcctatcgcggccgctggccgctcaaaaatggctggcctacggccaggcaatctaccagggc
gcggacaagccgcgccgtcgccactcgaccgccggcgcccacatcaaggcaccctgcctcgcgcgtttcggtgatgacggtg
aaaacctctgacacatgcagctcccggagacggtcacagcttgtctgtaagcggatgccgggagcagacaagcccgtcagg
gcgcgtcagcgggtgttggcgggtgtcggggcgcagccatgacccagtcacgtagcgatagcggagtgtatactggcttaac
tatgcggcatcagagcagattgtactgagagtgcaccatatgcggtgtgaaataccgcacagatgcgtaaggagaaaatacc
gcatcaggcgctcttccgcttcctcgctcactgactcgctgcgctcggtcgttcggctgcggcgagcggtatcagctcactcaaa
ggcggtaatacggttatccacagaatcaggggataacgcaggaaagaacatgtgagcaaaaggccagcaaaaggccagga
accgtaaaaaggccgcgttgctggcgtttttccataggctccgcccccctgacgagcatcacaaaaatcgacgctcaagtcaga
ggtggcgaaacccgacaggactataaagataccaggcgtttccccctggaagctccctcgtgcgctctcctgttccgaccctgcc
gcttaccggatacctgtccgcctttctcccttcgggaagcgtggcgctttctcatagctcacgctgtaggtatctcagttcggtgta
ggtcgttcgctccaagctgggctgtgtgcacgaaccccccgttcagcccgaccgctgcgccttatccggtaactatcgtcttgagt
ccaacccggtaagacacgacttatcgccactggcagcagccactggtaacaggattagcagagcgaggtatgtaggcggtgc
tacagagttcttgaagtggtggcctaactacggctacactagaaggacagtatttggtatctgcgctctgctgaagccagttacct
tcggaaaaagagttggtagctcttgatccggcaaacaaaccaccgctggtagcggtggtttttttgtttgcaagcagcagattac
gcgcagaaaaaaaggatctcaagaagatcctttgatcttttctacggggtctgacgctcagtggaacgaaaactcacgttaagg
gattttggtcatgcattctaggtactaaaacaattcatccagtaaaatataatattttattttctcccaatcaggcttgatccccagtaa
gtcaaaaaatagctcgacatactgttcttccccgatatcctccctgatcgaccggacgcagaaggcaatgtcataccacttgtccg
ccctgccgcttctcccaagatcaataaagccacttactttgccatctttcacaaagatgttgctgtctcccaggtcgccgtgggaaa
agacaagttcctcttcgggcttttccgtctttaaaaaatcatacagctcgcgcggatctttaaatggagtgtcttcttcccagttttcgc
aatccacatcggccagatcgttattcagtaagtaatccaattcggctaagcggctgtctaagctattcgtatagggacaatccgat
atgtcgatggagtgaaagagcctgatgcactccgcatacagctcgataatcttttcagggctttgttcatcttcatactcttccgagc
aaaggacgccatcggcctcactcatgagcagattgctccagccatcatgccgttcaaagtgcaggacctttggaacaggcagct
ttccttccagccatagcatcatgtccttttcccgttccacatcataggtggtccctttataccggctgtccgtcatttttaaatataggttt
tcattttctcccaccagcttatataccttagcaggagacattccttccgtatcttttacgcagcggtatttttcgatcagttttttcaattcc
ggtgatattctcattttagccatttattatttccttcctcttttctacagtatttaaagataccccaagaagctaattataacaagacgaa
ctccaattcactgttccttgcattctaaaaccttaaataccagaaaacagctttttcaaagttgttttcaaagttggcgtataacatagt
atcgacggagccgattttgaaaccgcggtgatcacaggcagcaacgctctgtcatcgttacaatcaacatgctaccctccgcga
gatcatccgtgtttcaaacccggcagcttagttgccgttcttccgaatagcatcggtaacatgagcaaagtctgccgccttacaac
ggctctcccgctgacgccgtcccggactgatgggctgcctgtatcgagtggtgattttgtgccgagctgccggtcggggagct
gttggctggctggtggcaggatatattgtggtgtaaacaaattgacgcttagacaacttaataacacattgcggacgtttttaatg
tactgaattaacgccgaattaattcgggggatctggattttagtactggattttggttttaggaattagaaattttattgatagaagt
attttacaaatacaaatacatactaagggtttcttatatgctcaacacatgagcgaaaccctataggaaccctaattcccttatctgg
gaactactcacacattattatggagaaactcgagcttgtcgatcgacagatccggtcggcatctactctatttctttgccctcggac
gagtgctggggcgtcggtttccactatcggcgagtacttctacacagccatcggtccagacggccgcgcttctgcgggcgattt
gtgtacgcccgacagtcccggctccggatcggacgattgcgtcgcatcgaccctgcgcccaagctgcatcatcgaaattgccgt
caaccaagctctgatagagttggtcaagaccaatgcggagcatatacgcccggagtcgtggcgatcctgcaagctccggatgc
ctccgctcgaagtagcgcgtctgctgctccatacaagccaaccacggcctccagaagaagatgttggcgacctcgtattgggaa
tccccgaacatcgcctcgctccagtcaatgaccgctgttatgcggccattgtccgtcaggacattgttggagccgaaatccgcgt
gcacgaggtgccggacttcggggcagtcctcggcccaaagcatcagctcatcgagagcctgcgcgacggacgcactgacgg
tgtcgtccatcacagtttgccagtgatacacatggggatcagcaatcgcgcatatgaaatcacgccatgtagtgtattgaccgatt
ccttgcggtccgaatgggccgaacccgctcgtctggctaagatcggccgcagcgatcgcatccatagcctccgcgaccggttgt
agaacagcgggcagttcggtttcaggcaggtcttgcaacgtgacaccctgtgcacggcgggagatgcaataggtcaggctct
cgctaaactccccaatgtcaagcacttccggaatcgggagcgcggccgatgcaaagtgccgataaacataacgatctttgtaga
aaccatcggcgcagctatttacccgcaggacatatccacgccctcctacatcgaagctgaaagcacgagattcttcgccctccga

gagctgcatcaggtcggagacgctgtcgaacttttcgatcagaaacttctcgacagacgtcgcggtgagttcaggctttttcatat
ctcattgccccccgggatctgcgaaagctcgagagagatagatttgtagagagagactggtgatttcagcgtgtcctctccaaat
gaaatgaacttccttatatagaggaaggtcttgcgaaggatagtgggattgtgcgtcatcccttacgtcagtggagatatcacat
caatccacttgctttgaagacgtggttggaacgtcttctttttccacgatgctcctcgtgggtgggggtccatctttgggaccactgt
cggcagaggcatcttgaacgatagcctttcctttatcgcaatgatggcatttgtaggtgccaccttccttttctactgtccttttgatg
aagtgacagatagctgggcaatggaatccgaggaggtttcccgatattaccctttgttgaaaagtctcaatagccctttggtcttct
gagactgtatctttgatattcttggagtagacgagagtgtcgtgctccaccatgttatcacatcaatccacttgctttgaagacgtg
gttggaacgtcttctttttccacgatgctcctcgtgggtgggggtccatctttgggaccactgtcggcagaggcatcttgaacgat
agcctttcctttatcgcaatgatggcatttgtaggtgccaccttccttttctactgtccttttgatgaagtgacagatagctgggcaat
ggaatccgaggaggtttcccgatattaccctttgttgaaaagtctcaatagccctttggtcttctgagactgtatctttgatattcttg
gagtagacgagagtgtcgtgctccaccatgttggcaagctgctctagccaatacgcaaaccgcctctccccgcgcgttggccga
ttcattaatgcagctggcacgacaggtttcccgactggaaagcgggcagtgagcgcaacgcaat

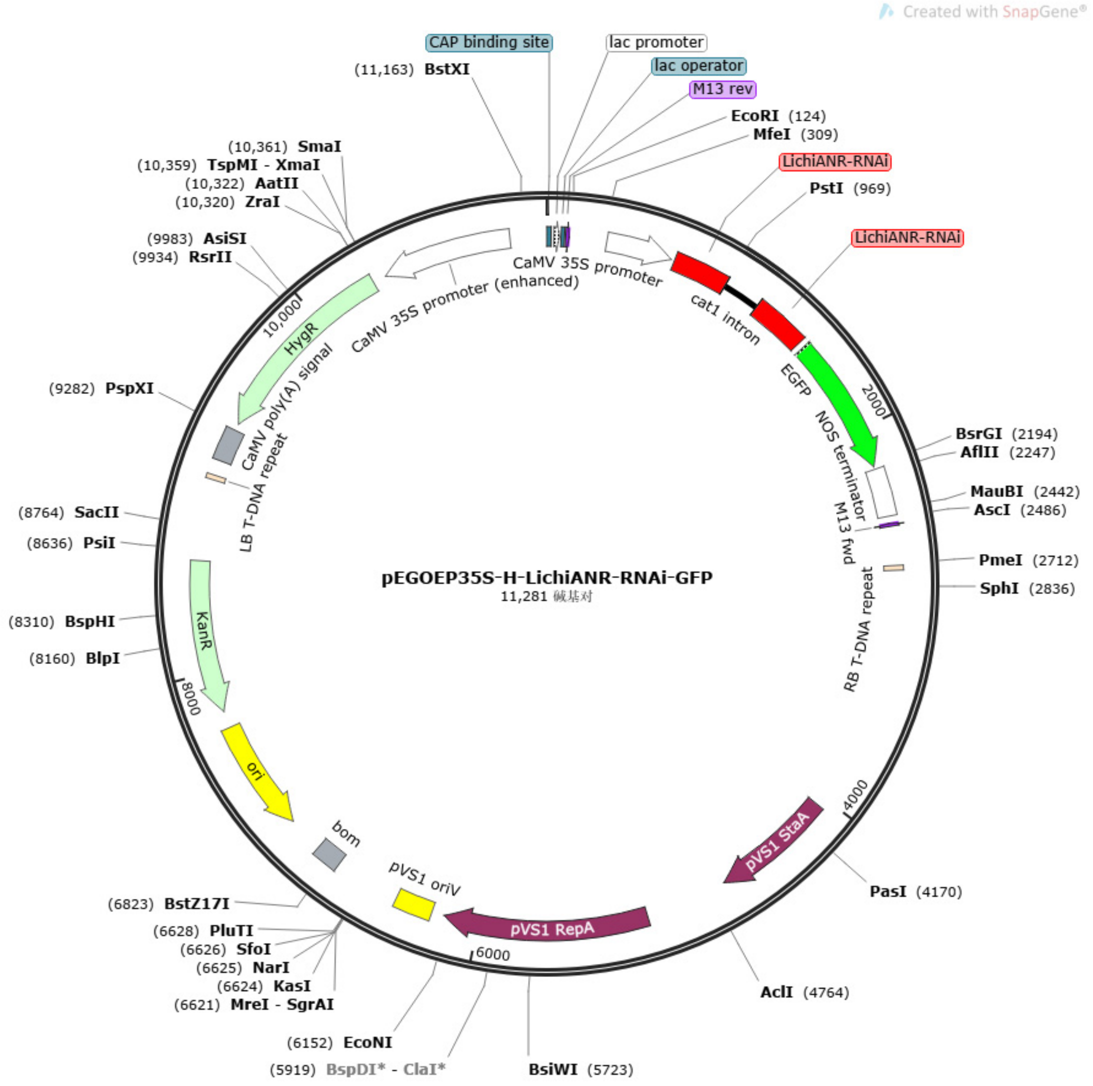

ANR-RNAi carrier map
